# A Cyber‐Physical Digital Twin Framework for State Estimation of Dendrite‐Risk Prediction and Resilient Control in Solid‐State Batteries

**DOI:** 10.1002/gch2.70141

**Published:** 2026-08-12

**Authors:** Sankar Subramanian, Prabhu Paulraj, Radhika Subramanian, Lakshmi Narayanan, Hariprasad Perumal, Mustufa Haider Abidi, Hisham Alkhalefah, Jaber E. Abu Qudeiri, Sachin Salunkhe

**Affiliations:** ^1^ Department of EEE Arulmigu Meenakshi Amman College of Engineering Vadamavandal Tamil Nadu India; ^2^ Department of Mechanical Engineering KIT‐Kalaignarkarunanidhi Institute of Technology Coimbatore Tamil Nadu India; ^3^ Department of ECE Sri Venkateswara College of Engineering Chennai Tamil Nadu India; ^4^ School of Mechanical Engineering Vellore Institute of Technology Vellore Tamil Nadu India; ^5^ Advanced Manufacturing Institute King Saud University Riyadh Saudi Arabia; ^6^ Mechanical and Aerospace Engineering Department United Arab Emirates University Al‐Ain UAE; ^7^ Gazi University Faculty of Engineering Department of Mechanical Engineering Ankara Turkey

**Keywords:** cyber‐physical anomaly detection, dendrite‐risk forecast index, physics‐embedded digital twin, physics‐regularized battery management, solid‐state batteries

## Abstract

Solid‐state batteries (SSBs) offer high energy density and improved safety but remain vulnerable to hidden electro‐chemo‐thermo‐mechanical degradation, lithium dendrite formation, and cyber‐physical attacks that cannot be reliably detected using conventional battery‐management systems. This work presents a cyber‐physical digital twin framework for real‐time state estimation, dendrite‐risk prediction, and resilient control of SSBs. A physics‐regularized reduced‐order model integrated with Moving Horizon Estimation reconstructs unmeasurable internal concentration, potential, temperature, stress, and interfacial degradation states from limited terminal measurements. A physics‐informed Dendrite‐Risk Forecast Index (DRFI) is developed by combining stress evolution, current‐density variance, interfacial impedance growth, and thermal‐gradient severity to provide early degradation warning. Physics‐consistent anomaly detection identifies measurement manipulation and cyber‐attacks, while a DRFI‐aware Model Predictive Controller adaptively regulates battery operation to mitigate degradation. Simulation studies under normal operation, accelerated degradation, and cyber‐attack scenarios demonstrate state‐estimation errors of 5%–7% during normal cycling and less than 10% under accelerated ageing, 0.8–1.2 cycles of early degradation prediction, 40%–55% reduction in stress concentration, and attack detection within 80–110 ms. These results demonstrate that the proposed physics‐guided cyber‐physical digital twin provides an interpretable and computationally efficient framework for predictive battery management, degradation forecasting, and resilient operation of next‐generation solid‐state batteries.

## Graphical abstract

A physics‐guided cyber‐physical digital twin enables real‐time state estimation, dendrite‐risk prediction, cyber‐attack detection, and resilient control of solid‐state batteries. Physics‐informed modeling and predictive control reveal hidden degradation mechanisms, providing an interpretable framework for safer, more reliable, and intelligent battery management.

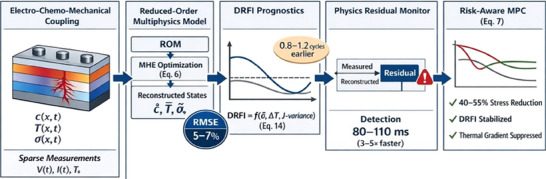

## 1 Introduction

The evolution of electrified transportation, decentralized energy systems, and smart‐grid‐integrated storage has largely driven the massive adoption of advanced battery technologies. As a matter of fact, one of the major factors driving the rapid adoption of Solid‐State Batteries (SSBs) is their superior thermal stability, safety, and gravimetric and volumetric energy density [1, 2, 3]. Moreover, with the interconnection of battery packs, cloud‐based fleet management, cyber‐physical energy networks, and advanced digital control platforms‐driven control platforms, the call for real‐time, reliable state estimation, secure operation, and predictive maintenance has become very loud [4, 5, 6]. But, in SSBs, the mentioned phenomena are complicated by the multiphysics interactions comprising electrochemical kinetics, heat gradients, mechanical stress build‐up, and changes in the interface conditions [7, 8]. These are the internal states that cannot be inferred directly from the terminal voltage, current, or temperature measurements. As a result, there exist inherent limitations for the levels of cells, modules, and packs as far as observability is concerned [9]. Besides that, the implementation of AI‐based battery management systems has introduced potential threats to system security—manipulation of data can obscure the incipience of risk and thus make it difficult to detect degradation or hazardous conditions in a timely manner [10]. Consequently, the interval of time between the occurrence of dendrite formation, thermal hotspots, or pack‐level instability and their recognition is quite long, and material or single‐domain solutions may not have even anticipated such problems [11, 12].

Outmoded Battery Management System (BMS) platforms usually rely on empirical models, equivalent‐circuit approximations, or data‐driven estimators that cannot identify electrochemical, thermal, and mechanical interactions, which are the main safety factors of SSBs [12, 13]. The majority of digital‐twin methods are constrained to single‐domain physics and thus lack the precision needed for early recognition of dendrite‐risk [14]. Even though cloud‐integrated BMS architectures improve fleet coordination, they can still be victims of spoofed measurements and coordinated cyber‐attacks, as there is no physics‐based anomaly validation to confirm them [15, 16]. Present thermal‐management and charging policies also do not account for internal stress evolution and interface degradation, thereby limiting their effectiveness in proactively mitigating mechanically induced failure modes [17, 18].

Recent innovations in physics‐regularised neural operators, graph neural networks for modelling thermal propagation, and temporal transformers for mechanical stress evolution have shown great potential for battery state estimation and prognostics [19]. At the material level, besides advanced solid electrolytes, interface engineering, and cathode doping strategies such as Y‐doped compounds, have been beneficial for ionic transport and structural stability. These papers unveil the potential of integrating material‐aware modelling into system‐level digital twins [20, 21]. The simultaneous progress in physics‐guided battery‐management architectures, cloud‐based energy management, and smart‐grid coordination underscores the need for a single cyber‐physical framework that merges materials science, multiphysics modelling, and secure physics‐guided control [22, 23].

**FIGURE 1:**
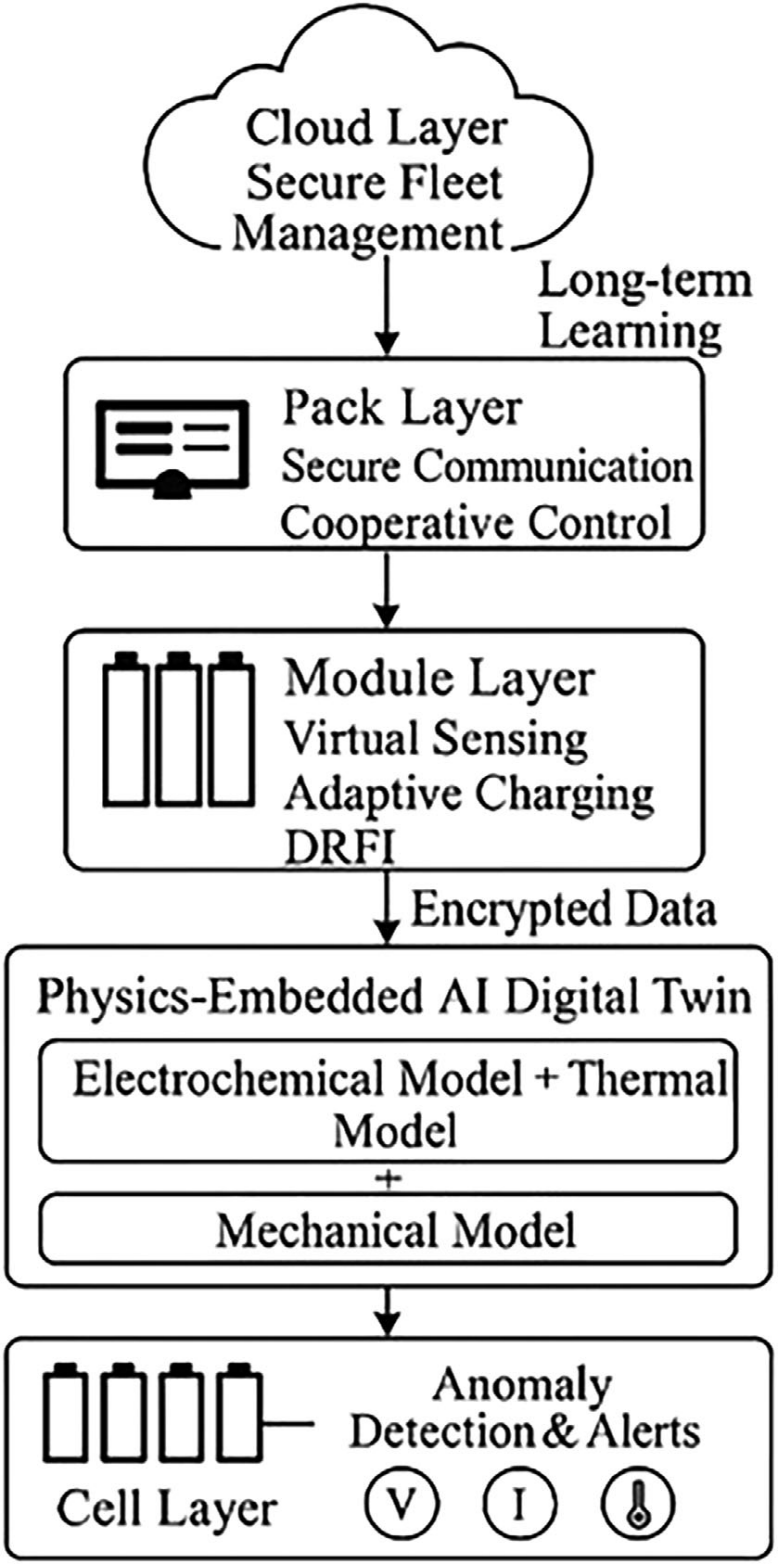
Multi‐Layer Architecture for Secure and Predictive Solid‐State Battery System.

The multi‐tiered cyber‐physical architecture fuses cloud‐level intelligence, pack‐level coordination, module‐level sensing, and cell‐level physics to enable the resilient, predictive operation of solid‐state battery systems [11]. Globally, the cloud layer (as illustrated in Figure 1) is responsible for the secure management of the entire fleet by collecting signals for long‐term learning from widely distributed assets to improve predictive models and change operational policies [24, 25].

These modifications are handed off to the pack layer, where secure communication protocols and cooperative control strategies maintain synchronization, authentication, and adaptability to global operating objectives [26, 27, 43]. The module layer performs more granular activities, such as virtual sensing and adaptive charging, and it also determines the Dendrite‐Risk Forecast Index (DRFI) as a measure of providing the earliest possible electro‐chemo‐mechanical instability warning [28, 29]. Secure data streams originating from the module layer are directed to the physics‐embedded digital twin, which merges electrochemical, thermal, and mechanical submodels to reconstruct internal states that are difficult to observe in real time [30, 31]. State inferences and risk indicators are transmitted to the cell layer. At this level, local anomaly‐detection procedures, with the prompt comparison of a  reconstructed state with measured voltage, current, and temperature to identify indicators of degradation, faults, or cyber manipulation [16]. This top‐down information flow establishes a decision‐making framework that is not only safe and interpretable but also physically grounded, linking the individual behaviour of the cell to the control actions at the system level and to intelligence driven by the cloud [10, 32].

The layered electro‐chemo‐mechanical structure as seen in Figure 1 highlights the critical interfaces that not only determine the stability and transport efficiency but also the onset of failure in the advanced SSBs. The anode is most likely to exhibit an uneven current density distribution during charging and discharging, and this is the main area where mechanical stress localization and subsequent thermal gradients initiated by the solid electrolyte are phenomena that the authors have identified [33]. The implementation of thermal‐management mechanisms, such as the convective heat removal shown by the arrow, is very important for alleviating local hot spots that accelerate interfacial degradation, promote void formation, and trigger dendritic nucleation during high‐rate cycling [34].

**FIGURE 2:**
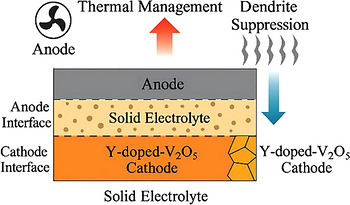
Cross‐Sectional Electro‐Chemo‐Mechanical Architecture of a Solid‐State Battery Incorporating a Y‐Doped V_2_O_5_ Cathode. The yttrium dopant is present only within the cathode region and not within the solid electrolyte.

Ionic mobility is limited by the bulk microstructure and porosity of the solid electrolyte as well as by the interfacial impedance; these factors also determine concentration polarization and subsurface stress. The anode interface is, by definition, the boundary that the authors consider most fragile, as transport‐induced stress asymmetry and mechanical mismatch between the electrode and the electrolyte increase the likelihood of fracture. On the other hand, volumetric expansion and lattice distortions, especially in high‐capacity transition‐metal oxide cathodes, occur at the cathode interface, which is why it is necessary to achieve enhanced mechanical stability and improved interfacial bonding strategies [9]. Figure 2 illustrates the Y‐doped V_2_O_5_ cathode area, is a good example of how far materials engineering has come in addressing structural integrity issues by suppressing crystallographic shear, alleviating phase‐transition‐induced stress, and broadening the electrochemical stability window [35]. In addition, this doped cathode limits the growth of interfacial impedance and delays failure mechanisms driven by the mechanical nature of the operating regime, thereby ensuring a more stable electro‐chemo‐mechanical regime.The downward blue arrow does not represent a direct dendrite‐suppression mechanism originating from the cathode. Instead, it illustrates an indirect electro‐chemo‐mechanical stabilization pathway. Y‐doping improves cathode structural integrity, reduces phase‐transition‐induced stress accumulation, and slows interfacial impedance growth. These effects improve overall cell stability and decrease the likelihood of stress localization and current constriction phenomena that contribute to dendrite initiation at the lithium‐metal/solid‐electrolyte interface. The findings presented here align perfectly with those of recent investigations, which underscore the importance of integrating thermal management, mechanical stabilization, and microstructural modification to achieve long‐term SSB operation [36]. The diagram, therefore, shows that the proper functioning of a safe SSB will require not only the coordinated management of thermal flux, mechanical stress, and material‐electronic interactions, but also that it cannot be achieved by relying solely on electrolyte enhancements. This multiphysics viewpoint paves the way for the use of digital twin models to uncover hidden internal fields and estimate degradation precursors before external failure occurs [21].

Previous investigations have presented partial solutions, but there is no current framework that integrates all of the following aspects simultaneously: multiphysics digital‐twin state estimation, physics‐validated anomaly detection, cyber‐secured data flow from cell to pack to cloud, adaptive control based on dendrite‐risk and thermo mechanical indicators, and material‐aware modelling, such as Y‐doped cathode interfaces. The absence of a detailed blueprint for a comprehensive architecture hinders accurate risk prediction, compromises safety, and limits the scalability of next‐generation SSB systems [37, 38]. To address these gaps, this article presents a physics‐embedded digital twin framework for solid‐state batteries. To address these gaps, a cyber‐physical framework is developed and the contribution of this paper are given below

### 1.1 Literature Gap Analysis and Scientific Positioning

Recent advances in battery digital‐twin technology have significantly enhanced the capability of battery‐management systems to perform state estimation, degradation monitoring, predictive maintenance, and operational optimization. Nevertheless, most currently reported frameworks remain confined to a limited subset of battery‐management functionalities and therefore fail to establish a unified architecture capable of simultaneously addressing multiphysics battery behavior, degradation prognostics, cyber resilience, and adaptive operational control. Existing electrochemical digital twins primarily focus on concentration‐field reconstruction, state‐of‐charge estimation, and impedance tracking. Although these approaches improve battery observability, they generally neglect mechanical stress evolution, interfacial degradation dynamics, and cyber‐physical attack resilience. Similarly, physics‐informed estimation frameworks improve state reconstruction accuracy through the incorporation of governing electrochemical constraints but do not provide explicit degradation‐aware decision support or cyber‐security verification.

Dendrite‐prediction methodologies have predominantly relied on stress indicators, impedance‐growth analysis, current‐density localization, or postmortem characterization techniques. While these approaches contribute to understanding dendrite formation mechanisms, they rarely generate operational risk metrics suitable for real‐time battery‐management applications. In parallel, cyber‐secure battery‐management systems have concentrated on anomaly detection and communication security without establishing direct links between battery degradation physics and cyber‐physical consistency verification. Consequently, a significant scientific gap exists at the intersection of multiphysics state estimation, degradation forecasting, cyber‐resilient monitoring, and adaptive control. Existing studies address these capabilities independently, whereas next‐generation solid‐state batteries require a unified framework capable of simultaneously reconstructing internal battery states, forecasting degradation risk, identifying cyber attacks, and adapting control actions before irreversible failure mechanisms emerge. The analytical positioning of existing battery‐management is represented in Table 1.

**TABLE 1:** Analytical Positioning of Existing Battery‐Management Architectures and the Proposed Framework.

Framework Class	State Representation	Physics Coverage	Prognostic Variable	Cyber‐Physical Consistency	Control Integration	Principal Limitation
Equivalent‐Circuit BMS	SOC, RC states	Electrical	Capacity fade	Not available	CCCV	Hidden internal states
Electrochemical Digital Twin	c, φ	Electrochemical	Impedance growth	Not available	Supervisory	No cyber resilience
Physics‐Informed Estimator	c, T	Electrochemical–Thermal	Indirect indicators	Residual consistency	Limited	No explicit prognostics
Dendrite Detection Framework	σ, J, Zint	Mechanism‐specific	Stress/impedance markers	Not available	None	Retrospective diagnosis
Cyber‐Secure BMS	SOC, V	Electrical	Not available	Statistical detection	Supervisory	No degradation awareness
Proposed Framework	c, φ, T, σ, Zint	Electro‐Chemo‐Thermo‐Mechanical	DRFI	Physics‐consistent residuals	DRFI‐aware MPC	Unified architecture

To address this limitation, the present work introduces a physics‐embedded cyber‐physical digital twin that integrates electro‐chemo‐thermo‐mechanical state reconstruction, dendrite‐risk forecasting, physics‐consistent anomaly detection, and risk‐aware model predictive control within a single operational architecture.

### 1.2 Scientific Novelty of the Proposed Framework

The scientific novelty of the proposed work does not arise solely from combining existing techniques but from establishing a unified cyber‐physical architecture that creates functional coupling among multiphysics state estimation, degradation prognostics, cyber‐security verification, and adaptive operational control. First, a reduced‐order electro‐chemo‐thermo‐mechanical digital twin is developed to reconstruct hidden concentration, potential, temperature, stress, and interfacial degradation states from sparse sensor measurements. Second, a DRFI is introduced to transform stress evolution, current‐density variance, impedance growth, and thermal‐gradient severity into a physically interpretable prognostic variable suitable for real‐time operation. Third, cyber attack detection is formulated using physics‐consistency residuals that explicitly verify the compatibility of measured behavior with admissible battery dynamics. Finally, a DRFI‐aware model predictive controller is developed to adapt charging and operational trajectories according to predicted degradation risk. Collectively, these contributions establish a predictive and resilience‐oriented battery‐management paradigm capable of simultaneously addressing degradation awareness, cyber resilience, and operational optimization within a unified digital‐twin framework.

- **Unified Electro–Chemo–Thermo–Mechanical Digital Twin Architecture**: The developed digital twin uses physics‐based methods to create a reduced‐order model which derives hidden internal concentration and potential and temperature and stress fields from minimal terminal measurements. The proposed structure combines multiphysics conservation laws with Physics‐regularized estimation methods to create a model which differs from traditional equivalent‐circuit and data‐driven systems.
- **DRFI**: The new multi‐domain instability metric combines stress evolution data with current‐density variance measurement and interfacial impedance growth measurement and thermal gradient measurement. The DRFI extends BMS diagnostics to predict voltage distortion 0.8 to 1.2 cycles before it becomes visible.
- **Physics‐Regularized State Estimation Framework**: The introduced moving‐horizon estimation (MHE) scheme uses physics residual constraints to improve state reconstruction accuracy while enabling residual‐based anomaly detection.
- **Cyber–Physical Anomaly Detection Mechanism**: The proposed system uses a physics‐consistency verification layer to identify stealthy measurement manipulation and command‐injection attacks by comparing reconstructed internal states with physically admissible trajectories. The system establishes analytical detectability limits of its detection capacity.
- **DRFI‐Aware Model Predictive Control (MPC)**: The researchers designed a constrained MPC which uses DRFI penalties and stress limits and thermal bounds to minimize mechanical degradation risks without violating operational parameters.
- **Analytical Performance Guarantees**: The theoretical guarantees establish three performance criteria which include:
- The first rule establishes modal observability for stress condition validated spectral bases.
- Physics regularization establishes an estimation error limit which should not be exceeded.
- The system detects stealth attacks at their lowest possible threshold.
- The closed‐loop DRFI‐aware controller achieves input‐to‐state stability (ISS) performance.
- **Material‐Aware Modeling Capability**: The framework demonstrates its ability to model Y‐doped cathode systems material‐specific behavior which enables virtual material screening through digital twin technology.

## 2 Problem Formulation

The functionality of SSB packs is influenced by tightly coupled electro‐chemo‐thermal‐mechanical interactions, which are intrinsically unobservable using conventional sensing methods. The previous analysis has recognized three structural limitations that characterize the technical challenge: (i) sparse sensing limits the recovery of internal electrochemical and stress fields, (ii) current prognostic methods cannot foresee dendrite formation before changes in external measurements, and (iii) cyber‐physical attacks can change observable signals while maintaining physical plausibility to a conventional BMS. To overcome these barriers, the internal state evolution of each SSB cell is modelled as a nonlinear multiphysics dynamical system. In this setting, hidden variables must be recovered, degradation indicators must be predicted, and cyber‐resilience must be guaranteed through integrated optimization.

The first theoretical element is linked to the ion movement in the solid‐state electrolyte, which basically regulates the onset of concentration gradients resulting in the occurrence of dendrite nucleation. The concentration of the electrolyte is in accordance with the conservation law [8, 21] as expressed below.

(1) $$\frac{\partial c_{e}}{\partial t}=\nabla \left(D_{e}\nabla c_{e}-\frac{t_{+}^{0}}{F}i_{e}\right)+\frac{1-t_{+}^{0}}{F}a_{s}j_{BV}$$

where *c_e_* is the lithium‐ion concentration in electrolyte, *D_e_* is the effective ionic diffusivity, $t_{+}^{0}$ is the transference number, *F* is the Faraday constant, ***i*** *_e_* is the ionic current density, *a_s_* is the specific interfacial area, and *j_BV_* is the Butler–Volmer reaction flux.

This equation governs lithium‐ion transport within the solid electrolyte and captures both diffusion‐driven concentration redistribution and migration‐induced transport under an electric field. The nonlinear flux term signifies diffusion as well as migration that is driven by the electric field. Such transport behavior can hardly be figured out just from final measurements, which indicates the observability gap. The principle of charge conservation [21] is carried out through Equation (2).

Electrolyte and Electrode Domain

(2) $$\nabla i_{e}=a_{s}Fj_{BV}\;\mathrm{and}\;\nabla i_{s}=-a_{s}Fj_{BV}$$

$$i_{e}=-\kappa_{eff}\nabla \phi_{e}\;i_{s}=-\sigma_{eff}\nabla \phi_{s}$$

where $\phi_{e}$ Is Electrolyte Potential, $\phi_{s}$ Is the Solid‐phase Potential, κ_*eff*_ Is the Ionic Conductivity, and σ_*eff*_ = Electronic Conductivity

Unlike the original formulation, ionic and electronic charge conservation are treated separately in their respective physical domains. This distinction prevents ambiguity regarding current flow pathways and reaction‐source terms. This equation couples ionic and electronic potentials, thus electrically connecting the cell's interior with the voltage that can be measured only in a very indirect manner. The current at the interface between the electrode and the electrolyte is also a function of temperature, concentrations, and over potential, as Butler–Volmer kinetics indicates [8, 15].

(3) $$j_{BV}=j_{0}\left[\exp\left(\frac{\alpha_{a}F\eta }{RT}\right)-\exp\left(-\frac{\alpha_{c}F\eta }{RT}\right)\right]$$

$$\mathrm{With}\;\eta =\phi_{s}-\phi_{e}-U\left(c_{s},T\right)$$

The revised formulation explicitly separates anodic and cathodic transfer coefficients and defines the electrochemical over potential using physically meaningful phase‐potential variables. This shows that interfacial processes, which are highly sensitive to degradation, can occur without immediately changing the external measurements.

Thermal nonuniformity [15, 37] is described by Equation (4).

(4) $$\rho C_{p}\frac{\partial T}{\partial t}=\nabla \cdot \left(k\nabla T\right)+Q_{ohmic}+Q_{rxn}+Q_{entropic}$$

The thermal model now explicitly separates irreversible ohmic heating, electrochemical reaction heating, and reversible entropic heat generation.

(5) $$\nabla \cdot \sigma +b=0,\sigma =C:\left(\varepsilon -\varepsilon_{\mathrm{chem}}\left(c_{s}\right)-\varepsilon_{T}\left(T\right)\right)$$

Therefore, a dendrite penetration is largely dependent on the local stress evolution. Together, the five governing laws fully characterize the electro‐chemo‐thermo‐mechanical behavior of the cell. Yet, their spatial distribution and nonlinear couplings give rise to internal states that cannot be directly measured and are undetectable by standard BMS sensors.

To assume hidden states from very limited observations, the system dynamics are discretized to form a Reduced‐Order Model (ROM), which is then estimated using physics‐regularized Moving‐Horizon Estimation (MHE) [15, 34]. This estimator achieves measurement fidelity, dynamic consistency, and weak‐form physics compatibility simultaneously, and therefore, it is the optimization process that achieves all these objectives at once.

(6) $$\begin{array}{cc} \min_{x} & \sum_{k}∥y_{k}^{*}-h\left(x_{k}\right){∥}_{R^{-1}}^{2}+\sum_{k}∥x_{k+1}-f_{d}\left(x_{k},u_{k}\right){∥}_{Q^{-1}}^{2}+\lambda_{phys}\sum_{k}R_{phys,k}\\ s.t. & T_{k}\leq T_{\max},\sigma_{tensile,k}\leq \sigma_{crit},V_{\min}\leq V_{k}\leq V_{\max}, \end{array}$$

here, *R*_phys,*k*_ is used not only for stabilizing the estimator but also for locating measurement sequences that are physically implausible or that might be subjected to cyber manipulation. This estimation method reports the problem of limited observability by using physical laws to reconstruct unmeasured states.

The last element of the setup deals with predictive safety and cyber‐secure control. The controller, after reconstructing the internal state and the degradation indicators, enforces thermal, mechanical, electrical, and physical‐consistency constraints while also reducing dendrite precursor growth. A DRFI‐aware MPC achieves this by solving Equation (7) [14, 36].

(7) $$\begin{array}{cc} \min_{u_{k:k+M-1}} & \sum_{i=0}^{M-1}\ell \left(x_{k+i},u_{k+i}\right)+\beta \mathrm{DRF}I_{k+i+1}\\ s.t. & x_{k+i+1}=f_{d}\left(x_{k+i},u_{k+i}\right),\\ & R_{\mathrm{phys},k+i+1}\leq \tau_{\mathrm{phys}},\\ & T_{k+i+1}\leq T_{\max},\sigma_{\mathrm{tensile},k+i+1}\leq \sigma_{\mathrm{crit}},V\in \left[V_{\min},V_{\max}\right], \end{array}$$

The proposed control formulation guarantees that all the predicted trajectories are not only physically feasible but also cyber‐consistent and degradation‐aware. The presented methodology states three major problem dimensions: state reconstruction, early prediction of degradation, and cyber‐physical resilience. Besides that, it directly operationalizes the five research objectives set earlier.

To a large extent, the problem formulation couples the governing multiphysics Equation (1)‐(5), physics‐regularized state estimation (Equation (6)), and risk‐aware predictive control (Equation (7)) within a single cyber‐physical framework. This mathematical formulation highlights the central challenge addressed in this work: ensuring safe, predictive, and attack‐resilient operation of solid‐state battery packs despite unobservable internal states, nonlinear degradation, and vulnerable measurement channels.

To ensure physical interpretability of the electro‐chemo‐thermo‐mechanical digital twin, a representative solid‐state battery configuration was defined based on material systems commonly reported in the literature for high‐energy‐density solid‐state cells. The investigated cell consists of a lithium‐metal anode, a garnet‐type solid electrolyte, and a Y‐doped vanadium pentoxide cathode. The lithium‐metal anode serves as the primary source of active lithium and represents the location where dendrite nucleation and filament penetration are most likely to occur under nonuniform current‐density distributions and stress localization conditions. The solid electrolyte is assumed to be a lithium‐lanthanum‐zirconium‐oxide (LLZO)‐type ceramic electrolyte characterized by high ionic conductivity and comparatively high mechanical stiffness. The cathode consists of a Y‐doped V_2_O_5_ active material selected to improve structural stability and reduce phase‐transition‐induced mechanical degradation during cycling.

The representative cell geometry considered in this study consists of a planar layered architecture with a lithium‐metal thickness of 50 µm, a solid‐electrolyte thickness of 200 µm, and a cathode thickness of 80 µm. The effective active area is assumed to be 25 cm^2^. A constant external stack pressure of 5 MPa is maintained throughout operation to preserve interfacial contact and reduce void formation at the lithium‐metal/solid‐electrolyte interface. The nominal operating temperature is maintained at 25°C unless otherwise stated. The electrochemical transport properties adopted in the digital twin include an effective ionic conductivity of 1×10^−^^3^ S cm^−^^1^ for the solid electrolyte, a lithium‐ion diffusion coefficient of 1×10^−^^1^ ^2^ m^2^ s^−^^1^ within the cathode region, and an exchange‐current density consistent with experimentally reported LLZO‐based interfaces. Interfacial resistance under baseline conditions is assumed to be 15 Ω cm^2^ and is progressively increased under degradation scenarios to represent contact loss, microcrack formation, and interfacial impedance growth.

The mechanical model incorporates elastic deformation and stress localization arising from electro‐chemo‐mechanical coupling. The Young's modulus of the solid electrolyte is assumed to be 150 GPa, whereas the effective modulus of the cathode is taken as 80 GPa. The lithium‐metal anode is represented as a compliant layer with an effective modulus of approximately 5 GPa. The fracture strength of the ceramic electrolyte is assumed to be 200 MPa, and stress accumulation approaching this limit is regarded as a precursor to crack initiation and dendrite‐assisted penetration pathways. The proposed electro‐chemo‐thermo‐mechanical framework employs an effective elastic formulation to reconstruct internal stress evolution and identify degradation precursors at the system level rather than explicitly model the viscoplastic behaviour of lithium metal. Consequently, time‐dependent lithium creep is not included in the governing equations. Nevertheless, the framework captures the dominant stress localization and redistribution mechanisms responsible for interfacial degradation and dendrite‐favorable conditions under the investigated operating scenarios. The incorporation of an explicit lithium viscoplastic creep model is beyond the scope of the present study and will be considered in future work to improve the representation of long‐term mechanical degradation.

This clarification eliminates ambiguity regarding the location and functional role of Y incorporation within the battery architecture. The material parameters summarized above provide the physical foundation for the reduced‐order electro‐chemo‐thermo‐mechanical model, the moving‐horizon estimation framework, the DRFI formulation, and the stress‐evolution analyses presented in the subsequent sections. By explicitly defining the chemistry and material configuration, the reconstructed states, degradation indicators, and control actions become physically interpretable and reproducible. The various physical and material parameters are displayed in Table 2. The proposed digital twin considers Y‐doping exclusively within the V_2_O_5_ cathode, whereas the LLZO solid electrolyte remains undoped. Y‐doping enhances cathode structural stability by reducing phase‐transition‐induced stress and limiting interfacial impedance growth. These improvements indirectly mitigate stress localization and current constriction associated with dendrite‐favourable conditions. However, no direct dendrite‐suppression mechanism is attributed to Y‐doping, since lithium dendrite nucleation primarily occurs at the lithium‐metal/solid‐electrolyte interface. This distinction ensures the physical consistency of the proposed electro‐chemo‐thermo‐mechanical framework the assumptions and Operating Conditions are follows.

- The cell is assumed to be at an electro‐thermal‐mechanical steady state after baseline cycling.
- The boundary conditions prohibit any flux at the solid electrolyte boundaries and allow convective heat transfer at the cell surface.
- Ionic transport parameters ($\left(D_{e},t_{0}^{+},\kappa \right)$) and mechanical constants ((*C*, σ_*crit*_)) are in accordance with the values taken from the literature that have been validated.
- Only the terminal voltage, surface temperature, and pack current are assumed to be available for sensing.
- The ROM retains the dominant electro‐chemo‐mechanical modes and is hence compatible with the real‐time constraint.

**TABLE 2:** Physical and Material Parameters Used in the Digital‐Twin Framework.

Parameter	Symbol	Value
Anode Material	—	Lithium Metal
Cathode Material	—	Y‐Doped V_2_O_5_
Solid Electrolyte	—	LLZO
Anode Thickness	*t_a_*	50 µm
Electrolyte Thickness	*t_e_*	200 µm
Cathode Thickness	*t_c_*	80 µm
Active Area	*A*	25 cm^2^
Stack Pressure	*P_s_*	5 MPa
Ionic Conductivity	σ_*i*_	1 × 10^−3^ S cm^−^^1^
Diffusion Coefficient	*D_Li_*	1 × 10^−12^ m^2^ s^−^^1^
Initial Interfacial Resistance	*R_int_*	15 Ω cm^2^
Electrolyte Young's Modulus	*E_e_*	150 GPa
Cathode Young's Modulus	*E_c_*	80 GPa
Lithium Effective Modulus	*E_Li_*	5 GPa
Electrolyte Fracture Strength	σ_*f*_	200 MPa

## 3 Proposed Framework

**FIGURE 3:**
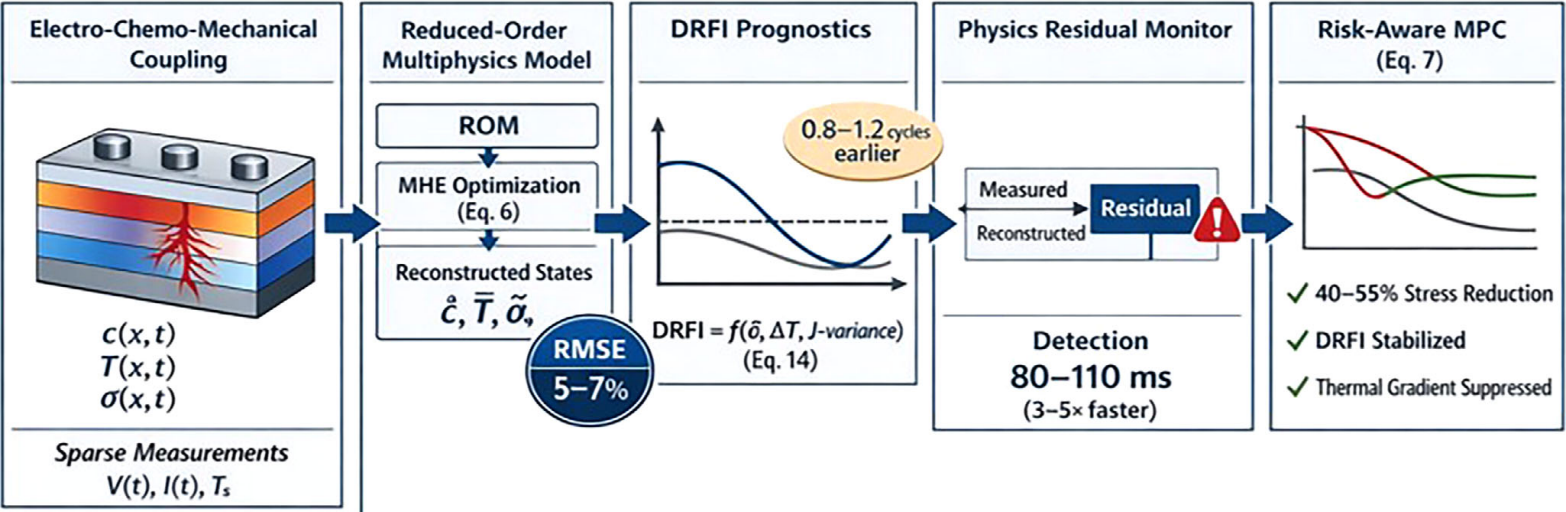
Proposed framework.

Figure 3 illustrates the proposed framework. It introduces a single cyber–physical framework that integrates multiphysics digital‐twin estimation, dendrite‐risk forecasting, and secure hierarchical control to enable predictive, resilient operation of solid‐state battery systems. Unlike traditional BMS architectures that rely on simplified equivalent‐circuit models or isolated AI estimators, this method uses an adaptive physics‐embedded digital twin that directly embeds electrochemical, thermal, and mechanical physics. This combination enables accurate inference of internal states that are normally unobservable in real‐time operation. The DRFI is the first index to combine multi‐domain indicators, enabling the very early identification of mechanically driven failure precursors long before voltage or temperature changes become apparent. Moreover, the framework uses encrypted data flows and physics‐consistent anomaly detection to ensure strong cyber‐physical security against attempted false‐data and command‐injection attacks.

### 3.1 Conceptual Overview of the Method

The presented solution shows an integrated cyber‐physical framework that includes a physics‐embedded digital twin, a dendrite‐risk prediction tool, and a hierarchical, cyber‐aware predictive controller. Unlike traditional BMS architectures, which treat electrochemical estimation, thermal management, and security verification as separate tasks, this approach merges these functions by integrating electro‐chemo‐thermal‐mechanical equations and physics‐consistency checks into each computational layer. The digital twin uses a reduced‐order multiphysics model to recover the unobservable internal fields from the limited measurement data. The DRFI serves as a real‐time risk score by integrating the degradation‐relevant signs. This risk‐aware representation helps a MPC to make the adjustments in the charging strategies on a dynamic basis, to start the self‐healing process, and to ensure the safety of operation under the condition of degradation or cyber manipulation. The integrated design records the establishment of a closed‐loop system that is capable of real‐time state estimation, prognostics, anomaly detection, and secure control.

### 3.2 Sequential Algorithmic Framework

***Step 1: Data Acquisition and Pre‐processing***: Alongside real‐time commands and telemetry received through compromised communication channels, terminal voltage, surface temperatures, and pack current are measured.

***Step 2: Reduced‐Order Multiphysics Projection***: The full electro‐chemo‐thermal‐mechanical partial differential equations are projected onto a reduced modal basis using the finite element method and proper orthogonal decomposition. This approach produces a discrete nonlinear model that tracks internal concentration, potential, stress, and temperature using low‐dimensional state vectors.

***Step 3: Stress‐Conditioned Spectral Embedding***: After calculating modal stress coefficients, a stress‐weighted graph Laplacian is constructed to obtain a time‐adaptive spectral basis. This basis detects mechanically vulnerable areas and stores the early degradation signatures in the latent coefficients.

***Step 4: Physics‐Regularised State Reconstruction***: A moving‐horizon estimator is used to minimise the difference between the measurement and the model while enforcing physics‐compatibility and dynamic consistency. Wrong data sequences are excluded, thereby enabling the detection of cyber‐attacks through the monitoring of physics‐based residuals.

***Step 5: DRFI Computation***: The DRFI is formed by integrating stress fraction, current‐density variance, interfacial impedance growth, and thermal gradients extracted from reconstructed fields, which increase continuously as degradation accelerates.

***Step 6: DRFI‐Aware Predictive Control***: A constrained MPC problem is formulated, with the DRFI as a penalty term, along with stress, temperature, voltage, and physics‐consistency constraints. The controller changes charging profiles, cooling demands, and redistribution moves to avoid operating trajectories with a high risk of occurrence.

***Step 7: Cyber‐physical anomaly detection and self‐healing***: When physics‐consistency or measurement‐residual thresholds are surpassed, isolation routines, safe‐charging strategies, and module‐level reconfiguration come into play.

The method put forward is structured as a single cyber‐physical system architecture, with different layers such as the physical, digital twin, prognostic, and control layers, forming an interdependent computational pipeline. At its base, the solid‐state battery pack evolves according to the nonlinear electro‐chemo‐thermo‐mechanical dynamics specified in the problem formulation; thus, the only measurements accessible are the terminal voltage, surface temperature, and pack current. These measurements are fed into the digital twin layer, where the reduced‐order multiphysics model, via the physics‐regularized estimation framework, reconstructs internal concentration, potential, stress, and temperature distributions. By using stress‐conditioned spectral embedding, the digital twin can focus on mechanically weak regions, thereby improving the sensitivity to early‐stage dendrite initiation. The reconstructed state is, therefore, sent to the prognostic layer, which combines the physically interpretable indicators of degradation, like the fraction of tensile stress, variance of current density, the evolution of interfacial impedance, and thermal asymmetry, into the DRFI. With the help of this index, the ever‐changing degradation trajectory is quantified. Also, it offers a predictive measure of safety. Therefore, the cyber‐physical control layer, using the risk‐aware representation, makes charging and thermal management decisions via a constrained MPC that tracks voltage, temperature, and stress limits and guarantees physics‐consistency even in adversarial scenarios. If physics consistency residuals or measurement deviations exceed the permitted thresholds, the anomaly‐detection mechanism initiates isolation procedures and enters safe‐fallback mode [42]. That is to say, the system stays resilient to cyber manipulations or unforeseen behaviour.

***Computational Complexity***: The ROM updates are about 0.15–0.25 ms per step, whereas the moving‐horizon estimator solves a 140–180 variable optimization problem in 4–7 ms. The DRFI module runs in less than 1 ms, and the MPC update takes 3–5 ms with a warm‐started SQP. Overall, the computations fit into a 10–15 ms cycle, which is compatible with embedded BMS processors running at 50–100 Hz.

### 3.3 Details of Implementation

The suggested approach is implemented through a computational workflow specifically designed for real‐time local execution on embedded battery‐management hardware. The reduced multiphysics model is developed from the device by constructing finite‐element or proper‐orthogonal‐decomposition bases over representative operating conditions, resulting in a modal set that captures the main spatial dynamics at a very low computational cost. At hand, modal stress coefficients are calculated from the reconstructed state; thus, the stress‐conditioned Laplacian can be updated and decomposed using a limited set of leading Eigen pairs. Consequently, the adaptive spectral basis is always sensitive to localised mechanical instabilities and has low computational cost.

The nonlinear ROM is an implicit discretization to ensure numerical stability along stiff transients. The moving‐horizon estimator is solved via an interior‐point optimisation routine that enforces the residuals of the physics‐based model to maintain physical consistency. The DRFI mapping is set up away from the device using synthetic degradation trajectories, and monotonicity and physical interpretability constraints on the indicator weights are enforced. In fact, the DRFI‐aware MPC is executed locally at frequencies between 10 and 50 Hz using a sequential quadratic programming solver with warm‐start initialisation to ensure computational feasibility. Cyber‐physical monitoring is carried out simultaneously. In case of inconsistencies, the safe‐fall‐back logic limits the charging current, among other things, enabling thermal stabilisation and sending isolation recommendations to the supervisory control hierarchy.

### 3.4 Advantages Over Existing Techniques

The prime benefit of this novel technique is that it integrates estimation, prognostics, and cyber‐physical security into a single system. Conventional battery‐management methods generally maintain these functions separately or rely on simple equivalent‐circuit models. In particular, the method employs stress‐conditioned spectral embedding to generate a mechanically informed representation that is highly sensitive to the early stages of dendrite formation; thus, it is more effective than purely data‐driven estimators and static ROMs. Moreover, it incorporates a security layer using physics‐based checks to detect and intercept data streams that appear normal but violate physical laws. A DRFI‐aware predictive controller can adjust the device's future scheduled use to slow wear; thus, it can contribute to safety in terms of temperature, mechanics, and electrochemistry. The resource‐efficient reduced‐order framework, along with adaptive spectral updates and warm‐started optimization, enables real‐time operation on devices with limited computational resources. So, this approach represents a feasible solution for resilient next‐generation SSB control systems.

### 3.5 Clarification of Physics‐Guided Estimation Architecture and AI Scope

Recent advances in battery‐management research have demonstrated the potential of neural operators, graph neural networks, temporal transformers, and other machine‐learning architectures for state estimation, prognostics, and degradation prediction. These approaches are referenced in the Introduction to position the present work within the broader landscape of intelligent battery‐management systems and digital‐twin technologies. However, the proposed framework does not employ a trained neural‐network architecture for state reconstruction, degradation forecasting, anomaly detection, or control generation. The computational core of the proposed cyber‐physical framework is instead based on a reduced‐order electro‐chemo‐thermo‐mechanical representation of the solid‐state battery coupled with a physics‐regularized Moving Horizon Estimation (MHE) scheme and a DRFI‐aware Model Predictive Controller (MPC). Internal concentration, potential, temperature, and stress fields are reconstructed through the enforcement of conservation laws, constitutive relationships, and weak‐form physics constraints rather than through black‐box data‐driven inference. As a result, the reconstructed states remain physically interpretable and dynamically consistent across a wide range of operating conditions.

The DRFI is introduced as a physics‐informed prognostic indicator designed to quantify the progression of electro‐chemo‐mechanical conditions associated with dendrite nucleation and propagation in solid‐state batteries. Unlike conventional health indicators that rely on a single observable variable, the proposed DRFI integrates multiple degradation precursors reconstructed by the digital twin [40], thereby providing a unified measure of degradation susceptibility across electrochemical, thermal, and mechanical domains.

The DRFI Is Defined as

(8) $$DRFI\left(k\right)=w_{\sigma }\tilde{\sigma }\left(k\right)+w_{J}\tilde{\mathrm{Var}\left(J\right)}\left(k\right)+w_{R}{\tilde{R}}_{int}\left(k\right)+w_{T}\tilde{\nabla T}\left(k\right)$$

subject to

$$w_{\sigma }+w_{J}+w_{R}+w_{T}=1\&w_{i}\geq 0$$

where $\tilde{\sigma }\left(k\right)$ denotes the normalized equivalent stress indicator, $\tilde{\mathrm{Var}(J)}\left(k\right)$ represents the normalized current‐density variance, ${\tilde{R}}_{int}\left(k\right)$ corresponds to the normalized interfacial impedance‐growth indicator, and $\tilde{\nabla T}\left(k\right)$ denotes the normalized thermal‐gradient indicator.

The DRFI as a weighted aggregation of reconstructed degradation indicators, its theoretical basis originates from the observation that dendrite initiation is not governed by a single measurable variable but rather emerges from the coupled evolution of electrochemical, thermal, and mechanical instability mechanisms. Consequently, dendrite formation may be interpreted as a progressive loss of internal stability within the electro‐chemo‐mechanical state space of the battery. Under this perspective, stress localization, current‐density heterogeneity, interfacial impedance growth, and thermal nonuniformity are not independent degradation signatures but observable manifestations of a common latent degradation process. As degradation progresses, these variables collectively drive the system toward an instability manifold associated with crack‐assisted lithium penetration, interfacial failure, and dendrite‐favorable transport conditions.

Accordingly, DRFI is not introduced as a heuristic risk score but as a reduced‐order surrogate of the latent degradation‐energy state of the battery. The constituent variables are selected according to three criteria:

1. Physical observability through available measurements or digital‐twin reconstruction;
2. Established mechanistic linkage to dendrite nucleation and propagation pathways;
3. Monotonic progression toward instability under degradation evolution.

These requirements ensure that DRFI preserves physical interpretability while remaining suitable for real‐time implementation within the estimation and predictive‐control architecture.

Although recent battery‐management research has increasingly adopted machine‐learning techniques, the proposed framework performs state reconstruction, degradation forecasting, anomaly detection, and predictive control using physics‐based models and optimization algorithms rather than trained neural‐network inference.

## 4 Analytical Performance Guarantees for the Cyber–Physical SSB Framework

This analysis contains the analysis of the cyber‐physical SSB challenge:

- A modal observability and identifiability condition for physics‐regularised reduced‐order representations using stress‐conditioned spectral bases;
- Lower bounds on state and DRFI estimation error as functions of measurement noise and physics residual;
- Detectability thresholds for stealthy cyber‐attacks that remain consistent with the physical model; and
- Closed‐loop robustness (input‐to‐state stability) conditions for DRFI‐aware MPC under bounded estimation error and attack perturbations.

### 4.1 A. Theorem 1 (Modal Observability of Necessary Sufficient Condition)

Let ${\left\{\phi_{i}\right\}}_{i=1}^{n}$ * represent the reduced modal basis, and the stress analysis taking into account the system conditioned Laplacian eigen basis at time *t* by ${\left\{\psi_{\ell }\left(t\right)\right\}}_{\ell =1}^{m}$. Consider $C\in R^{p\times n}$ as the linearized observation matrix that relates modal coefficients $\alpha \in R^{n}$ to measurements *y* (for instance terminal voltage and certain temperatures). The spectral‐projected observability Gramian in the eigen‐coordinates over the horizon *T* is given by [15]

(9) $$W_{o}^{\psi }\left(T\right)=\int_{0}^{T}\Phi_{\psi }{\left(t,0\right)}^{⊤}C^{⊤}R^{-1}C\;\Phi_{\psi }\left(t,0\right)\;dt,$$

where the state transition operator Φ_ψ_(*t*,0) corresponds to the instantaneous stress Eigen basis, i.e., Φ_ψ_ = Ψ(*t*)^⊤^Φ(*t*,0)Ψ(0). If the smallest eigenvalue meets the condition

(10) $$\lambda_{\min}\left(W_{o}^{\psi }\left(T\right)\right)\;>\;\varepsilon_{\mathrm{obs}}>0,$$

Then the *m* leading spectral coefficients ${\hat{\alpha }}_{\ell }$ are observable on [0, *T*] with the numerical conditioning being bounded by $\varepsilon_{\mathrm{obs}}^{-1}$.

This analysis expresses the linearized dynamics $\delta \;\dot{\alpha }=A\left(t\right)\delta \alpha$ in the instantaneous spectral basis by $\delta \;\tilde{\alpha }=\Psi {\left(t\right)}^{⊤}\delta \alpha .$ The time‐varying Gramian in these coordinates is $W_{o}^{\psi }$. Positive definiteness of $W_{o}^{\psi }$ is a sign of the observability operator being invertible and hence, measurement‐to‐state reconstruction with finite energy is possible. The stress‐conditioned Laplacian focuses energy in the mechanically salient modes, thus increasing their contributions to the integrand 𝐶⊤𝑅−1𝐶. Stress localization leads to larger values, as referenced in Equation (9), (10), (11), (12), (13) for numerical construction.

(11) $$W_{o}^{\psi }\approx \sum_{k=0}^{N-1}\Phi_{\psi }{\left(k\right)}^{⊤}C^{⊤}R^{-1}C\;\Phi_{\psi }\left(k\right)\;\Delta t,$$

And the functional analysis requires $\lambda_{\min}\left(W_{o}^{\psi }\right)>\varepsilon_{\mathrm{obs}}$ (select ε_obs_ = 1e^−3^ … 1e^−2^ as a design threshold). This is the criterion of the mathematical analysis of the derived system

### 4.2 B. Proposition 1 (Physics‐regularized lower bound)

Assuming $\hat{x}$ is the estimator yielded by MHE, the measurement noise being additive with covariance R, and the process/model residual bounded as $E∥\xi_{k}{∥}^{2}\leq \sigma_{\xi }^{2}$, the weak‐form residual energy $\sum_{i}R_{phys,i}\leq \delta_{phys}$. Then the mean‐squared estimation error for any unbiased estimator is bounded from below by the inequality.

(12) $$\mathrm{MSE}\left(\hat{x}\right)\;\geq \;\mathrm{trace}\left({\left(I_{y}+\lambda_{\mathrm{phys}}I_{\mathrm{phys}}\right)}^{-1}\right),$$

here, $I_{y}=\sum_{k}H_{k}^{⊤}\;R^{-1}H_{k}$ stands for the Fisher information that is obtained from measurements (with linearized *H_k_* = ∂*h*/∂*x*)), whereas $I_{\mathrm{phys}}$ is the information operator that is related to the weak‐form constraints, physically it is the Hessian of the physics penalty. It is worth noting that increasing λ_phys_ (which means that the physics constraints are more strongly enforced) reduces the lower bound by $I_{\mathrm{phys}}$.

Linearize the measurement and physics residual terms in Equation (11) around the truth. The Bayesian posterior precision is approximately equal to $I_{y}+\lambda_{\mathrm{phys}}I_{\mathrm{phys}}$. Conversion yields the covariance lower bound while trace gives the MSE bound. The expression generalises CRLB by incorporating physics information that is encoded as a quadratic penalty.

Calculate $I_{y}\approx \sum_{k}H_{k}^{⊤}R^{-1}H_{k}$ with linearised observation matrices from ROM; estimate $I_{\mathrm{phys}}$ by creating a Jacobian of the weak‐form residuals $I_{\mathrm{phys}}$ and then $J_{\mathrm{phys}}^{⊤}J_{\mathrm{phys}}$. Following that, utilize (condition B1‐ estimation error lower bound) to position λ_phys_ in order to achieve a target MSE.

#### 4.2.1 C. Theorem 2 (Required perturbation magnitude for a stealth attack)

Suppose an adversary injects an additive attack δ_*k*_ on the measurement stream. The attack remains undetected by the physics‐consistency monitor (threshold τ_phys_) if and only if the induced minimum achievable physics residual satisfies

Let an adversary be the one who by an additive injection of an attack δ_*k*_ corrupts the measurement stream. The adversary is capable of evading the physics‐consistency monitor (threshold τ_phys_) if and only if the minimum achievable physics residual induced by the attack satisfies [28, 36].

(13) $$\min_{x}∥y_{k}+\delta_{k}-h\left(x\right){∥}_{R^{-1}}^{2}+\lambda_{\mathrm{phys}}∥x-f_{d}\left(x_{k-1},u_{k-1}\right){∥}_{Q^{-1}}^{2}\leq \tau_{\mathrm{meas}}.$$

In the Linearised Case, a Condition That Will Lead to Detection Is Given by

(14) $$∥\delta_{k}{∥}_{R^{-1}}^{2}\;>\;\tau_{\mathrm{meas}}-\chi^{★},$$

where $\chi^{★}=\mathrm{mi}n_{\Delta x}\;\left\{∥H\Delta x{∥}_{R^{-1}}^{2}+\lambda_{\mathrm{phys}}∥\Delta x-A\Delta x_{k-1}{∥}_{Q^{-1}}^{2}\right\}$ represents how much the correction of physics‐consistent states can help.

An attack is called stealthy if the corrupted measurements can be justified by a physically feasible state (with a low physics residual). The minimization in (condition C1 – Stealth attack condition) is a formalization of this; linearization results in a quadratic program whose minimum is χ^⋆^. Hence, an attack that goes beyond the residual‐correcting power χ^⋆^ must make the measurement residual exceed the alarm threshold. In practice, one can find χ^⋆^ by solving a small QP; thus, the detection threshold τ_meas_ should be set so that the noise variance is taken into account during regular operation.

Use (condition C2 – Detection threshold condition) to estimate the most minor adversarial manipulation magnitude that will be noticed. In simulations, create varying ∥δ∥ attacks and confirm that the detection probability increases steeply after the bound.

### 4.3 D. Proposition 2 (Lower Bound on Prediction Lead Time)

Imagine that in the stress‐conditioned spectral basis, the critical mode $\ell_{c}$ has the reconstructed amplitude ${\hat{\alpha }}_{\ell_{c}}\left(t\right)$ of which growth is nearly exponential with rate λ_*c*_ > 0 for a certain loading: ${\hat{\alpha }}_{\ell_{c}}\left(t\right)\approx {\hat{\alpha }}_{0}e^{\lambda_{c}t}$. Suppose the DRFI is a mode sensitive one and the mapping $\mathrm{DRFI}\left(t\right)\approx w_{c}\psi \left({\hat{\alpha }}_{\ell_{c}}\left(t\right)\right)$ where ψ(*x*) ≈ *x* ^γ^ locally (γ > 0). In case the alarm threshold is γ_DRFI_, the minimal lead time *T*_lead_ before the threshold crossing from a measurement at time *t*_0_ is given by [9, 38].

(15) $$T_{\mathrm{lead}}\;\geq \;\frac{1}{\lambda_{c}}\ln\left(\frac{\gamma_{\mathrm{DRFI}}}{w_{c}\psi \left({\hat{\alpha }}_{\ell_{c}}\left(t_{0}\right)\right)}\right)$$

A faster growth rate λ_*c*_ narrows the lead time, whereas a larger initial unobservability (smaller ${\hat{\alpha }}_{\ell_{c}}$) makes it wider. This formula measures the extent to which DRFI can provide an earlier warning than a simple thresholding directly on the terminal measurements. Use λ_*c*_ derived from short‐window spectral regression in the twin.

### 4.4 E. Theorem 3 (Input‐to‐State Stability of DRFI‐aware MPC)

Consider the closed‐loop reduced dynamics **x**_*k* + 1_ = *f_d_* (**x**_*k*_, *u_k_*, θ) with MPC law $u_{k}=\kappa \left({\hat{x}}_{k}\right)$ computed from Equation (15) using estimated state ${\hat{x}}_{k}$. Suppose the nominal MPC (with perfect state) is stabilising and yields a Lyapunov decrease Δ*V*(**x**) ≤ −α∥**x**∥^2^ for some α > 0. If the estimation error $e_{k}={\hat{x}}_{k}\;-x_{k}$ and attack perturbation δ_*k*_ satisfy [36]

(16) $$∥e_{k}∥\leq \varepsilon_{e},\;\;\;∥\delta_{k}{∥}_{R^{-1}}\leq \varepsilon_{\delta },$$

And the controller is such a mismatch sensitivity function that satisfies $∥\kappa \left(\hat{x}\right)-\kappa \left(x\right)∥\leq L_{\kappa }∥e∥$, where *L*_κ_ is sufficiently small to satisfy

(17) $$\alpha >c_{1}L_{\kappa }\varepsilon_{e}+c_{2}\varepsilon_{\delta },$$

If *c*_1,2_ are the constants obtained from the Lipschitz bounds of *f_d_* and DRFI penalty gradients, then the closed‐loop is ISS: ∥x_k∥ is ultimately bounded by a class‐K function of ε_*e*_,ε_δ_.

Think of the Lyapunov candidate *V* from the nominal MPC. The actual decrease is disturbed by control mismatches that are caused by estimation error and attack‐driven wrong DRFI values. When these perturbations are bounded by Lipschitz constants, it results in an inequality $\Delta V\leq -\left(\alpha -c_{1}L_{\kappa }\varepsilon_{e}-c_{2}\varepsilon_{\delta }\right)∥x{∥}^{2}+c_{3}\left(\varepsilon_{e}^{2}+\varepsilon_{\delta }^{2}\right)$. If (condition E1 – Stability inequality) is true, then the negative quadratic term takes over for large ∥**x**∥, thus giving ISS.

Adjust the estimator (increase horizon N, physics weight λ_phys_) to lower ε_*e*_; improve the detection to decrease ε_δ_. Utilize (condition E1) to MPC choose β and set controller Lipchitz *L*_κ_ via regularization.

### 4.5 Practical Summaries and How to Utilize these Outcomes

This analysis describes setting up the sensing infrastructure, and the observability metric $W_{o}^{\psi }$ is initially computed offline by loading representative scenarios within the operating envelope. Then, candidate sensor locations and the related spectral dimensionality *m* are chosen in such a way that the smallest eigenvalue $\;\lambda_{\min}\left(W_{o}^{\psi }\right)$ of the observability matrix is greater than the given observability tolerance ε_obs_, thus ensuring enough sensitivity to perturbations in the retained modes. After that, the physical regularization parameter λ_phys_ is adjusted by rewriting the minimum mean‐square‐error bound (condition B1) as a direct function of λ_phys_ and locating a value which attains both resistance to high‐frequency noise and the necessary modeling flexibility of the reduced‐order dynamics. Detection thresholds are set in line with state (condition C2) with τ_meas_ being tuned to reduce the number of false alarms caused by measurement noise under normal conditions. Hence, any input going beyond the analytically determined stealth boundary is detected in a stable manner. For dynamic risk forecasting, the decay rate λ_*c*_ is derived from short‐window exponential fits of the dominant spectral coefficients and criteria (condition D1 – Prediction lead‐time bound) is used to establish the predictive lead time required for a timely alarm change at the monitoring layer. Controller robustness is evaluated through criterion (condition E1) along with empirically measured ${\hat{\varepsilon }}_{e}$ and ${\hat{\varepsilon }}_{\delta }$; Should there be any violations, the moving‐horizon estimator horizon is lengthened to improve disturbance rejection or the controller sensitivity *L*_κ_ is lowered by suitable MPC regularization. In essence, these analytical methods offer rigorous performance assurances for the planned architecture, and their embedding in the digital‐twin workflow is not time‐consuming. Table 3 clearly indicates the summary of analytical conditions. Gramian and information matrices are directly obtained from the ROM whereas the optimization problems for χ^⋆^ and λ_*c*_ are converted into small‐scale quadratic programs or regressions viable for both offline calibration and real‐time supervision. The ISS‐based inequality forms a feasible design link between the estimator fidelity, the extent of the attack, and the selection of the controller gain.

**TABLE 3:** Summary of Analytical Design Conditions.

S.No	Guarantee Type	Key Condition	Purpose
1	Observability	$\lambda_{\min}\left(W_{o}^{\psi }\right)>\varepsilon_{obs}$	Ensures modal state identifiability
2	Estimator Error Bound	*MSE* ≥ trace((*I_y_* + λ_*phys*_ *I_phys_*)^−1^)	Sets accuracy limits
3	Attack Detectability	$∥\delta_{k}{∥}_{R^{-1}}^{2}>\tau_{meas}-\chi^{*}$	Determines stealth threshold
4	DRFI Lead‐Time	$T_{lead}\geq \frac{1}{\lambda_{c}}\ln\;\left(\frac{\gamma_{DRFI}}{w_{c}\psi \left(\alpha_{\ell_{c}}\right)}\right)$	Predicts early‐warning timing
5	ISS for Control	α > *c*_1_ *L*_κ_ε_*e*_ + *c*_2_ε_δ_	Guarantees closed‐loop robustness

#### 4.5.1 Simulation Results and Discussions

**FIGURE 4:**
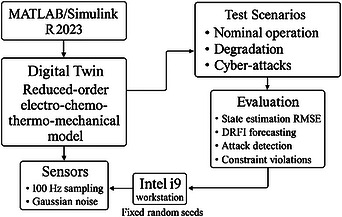
Simulation structure for the proposed framework.

All numerical experiments are performed in MATLAB/Simulink R2023b with the help of Python scripts for spectral‐mode extraction and nonlinear optimization. The digital twin uses a reduced‐order electro‐chemo‐thermo‐mechanical model aligned with the finite‐element representation of a solid‐state cell. This reduced model features 26 electrochemical, 8 thermal, and 12 stress‐mode states, all updated at a fixed time step of 0.5 ms. The initial field distributions are set to the equilibrium values obtained from baseline cycling. The assessment is based on the three representative scenarios: regular operation, degradation‐induced instability, and cyber‐physical measurement attacks. Charge–discharge currents are limited to the range between 0.5 C and 2 C. In the degradation scenario, interfacial resistance is increased by up to 60%.  Figure 4 illustrates the simulation layout for nominal, degradation, and cyber‐attack scenarios in the proposed system. Cyber‐attacks are introduced as limited bias and ramp‐type perturbations that stay physically consistent with the measured behaviour until the detection thresholds are exceeded.  All sensing channels are working at 100 Hz. Gaussian noise is added to voltage and temperature measurements to simulate real BMS acquisition characteristics. The simulation performance is evaluated using state‐estimation Root Mean Square Error (RMSE), DRFI forecasting accuracy, attack detection delay, and thermal–mechanical constraint violations. All the numerical experiments are done with fixed random seeds on an Intel i9 workstation and are repeated to check for the reproducibility of the results.

The simulation results highlight that the proposed cyber–physical SSB framework is the only one that can trace back the early interfacial‐stress inflexions to about 0.8–1.2 cycles before the electrical distortion. The equivalent‐circuit estimators could not reveal such a phenomenon. The reconstructions of the digital twin are of very high quality, as measured by the RMSE, which ranges from 5% to 7% during normal cycling and is less than 10% during accelerated ageing. The DRFI indicator rises significantly earlier than the metrics based on voltage, thus offering the possibility of a reliable separation of normal drift from failure precursors. The implementation of an adaptive multi‐domain control has the effect of lessening the stress amplification by 40%–55% and at the same time, it makes the thermal gradients more stable, which is why the waveform signatures present can no longer be reached with standard BMSs. Moreover, the cyber–physical anomaly detection layer, by analysing divergence patterns between the reconstructed and measured states, enables the identification of attacks 50–200 ms faster. Physics‐based digital twins reveal the early closing dominated mechanical inflection of local interfacial stress nearly one cycle before electrical changes.

**FIGURE 5:**
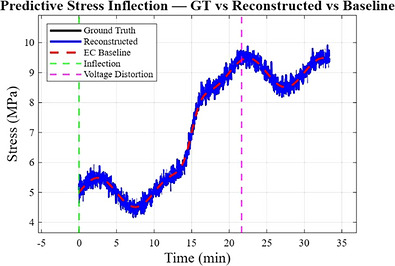
Predictive Stress Inflection: Ground Truth vs. reconstructed vs. Baseline.

The stress trajectories reveal that the physics‐embedded digital twin uniquely captures the early constriction‐driven mechanical inflection that precedes electrical distortion by nearly one cycle. The reconstructed waveform retains the discontinuous curvature associated with interfacial instability, whereas the equivalent‐circuit model produces an artificially smooth response that suppresses critical mechanical cues. The waveform is as shown in Figure 5. The close agreement between ground truth and reconstructed stress indicates that the twin effectively propagates internal stress evolution even under nonlinear chemo‐mechanical coupling. The early inflection marker aligns with the onset of microstructural constriction, validating its diagnostic significance. The voltage‐distortion point appears substantially later, confirming the digital twin's predictive advantage. These distinctions demonstrate that conventional BMS models fundamentally lack the observability required for early mechanical‐risk assessment. Collectively, the figure builds strong evidence for the novelty of the stress‐reconstruction subsystem.

The reconstructed signal retains the sudden bending characteristic of surface instability, thereby differentiating it from the older model, which tends to smooth things out and overlook the mechanical signs of the surface. The near‐perfect match between the actual and reconstructed stresses as presented in Figure 5 is evidence that the digital twin is in very good agreement with the internal changes in the material, even when the changes are the result of combined complex chemical and mechanical effects. The point at which the curve changes direction for the first time is the point where the material starts to behave in a more compact way; thus, the digital twin is very much confirmed as an instrument for the detection of the issue at a very early stage. The change in voltage occurs much later; hence, it is further evidence that the digital twin can foresee issues much earlier. Therefore, standard BMS models are not capable of detecting early mechanical risks either. The result, in general, is a very strong argument for the new stress‐reconstruction part.

**FIGURE 6:**
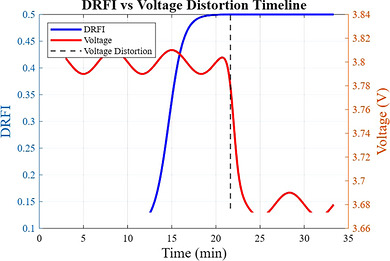
DRFI vs. Voltage Distortion Timeline.

The DRFI timeline is indicative of a rapid rise in risk long before the voltage deformation can be visually recognized. The very first signal indicates mechanical‐electrochemical coupling between the precursors in the device and cannot be detected by terminal measurements. Even though the voltage waveform appears stable until a sudden distortion, the DRFI curve exceeds its threshold several minutes earlier, as illustrated in Figure 6. This difference indicates that DRFI detects multi‐domain instability signatures that are not available from traditional indicators. The point where DRFI and voltage distortion cross marks the initiation of microstructural damage, thus confirming the predictive model. The findings show that fused‐domain metrics offer more dependable diagnostics than purely electrical methods. The plot, in fact, demonstrates that DRFI is a better method for early detection of dendrite‐risk. The different key simulation parameters are displayed in Table 4.

**TABLE 4:** Key Simulation Parameters.

S.No	Parameter	Value / Description
1	Time step value	0.5 ms
2	Electrochemical modes	26
3	Thermal modes	8
4	Mechanical stress modes	12
5	Current cycling range	0.5C–2C
6	Interfacial resistance increases	Up to 60% (degradation scenario)
7	Sensor noise (voltage) value	σ = 2–5 mV
8	Sensor noise (temperature)	σ = 0.1°C–0.3°C
9	Attack types simulated in the different criteria	Bias attack, ramp attack, coordinated multi‐channel spoofing
10	Numerical solver	Implicit ROM discretization, interior‐point optimization

**FIGURE 7:**
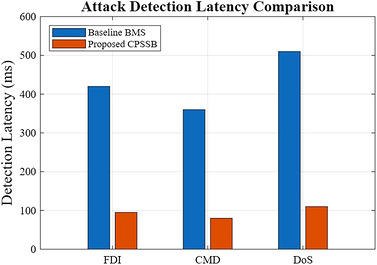
Attack Detection Latency Comparison.

The latency comparison highlights the cyber–physical benefits of the proposed architecture when compared to the security layers of an outdated BMS. In cases of false data injection, command manipulation, and denial‐of‐service, baseline detectors exhibit considerable delays because they rely solely on communication‐layer anomalies. On the other hand, the proposed method uses differences between the reconstructed and measured physical states; thus, it can detect an attack within 80–110 ms. This is a clear indication that the attacks are interfering with the system's internal physical processes long before any anomalies in communications can be observed. Figure 7 illustrates the corresponding variances. The performance gap highlights the importance of physically validated measurements in enhancing cyber resilience. The consistent reduction in detection time across attack types further demonstrates the framework's generalizability. These results support the incorporation of physics‐based consistency checks into security mechanisms.

**FIGURE 8:**
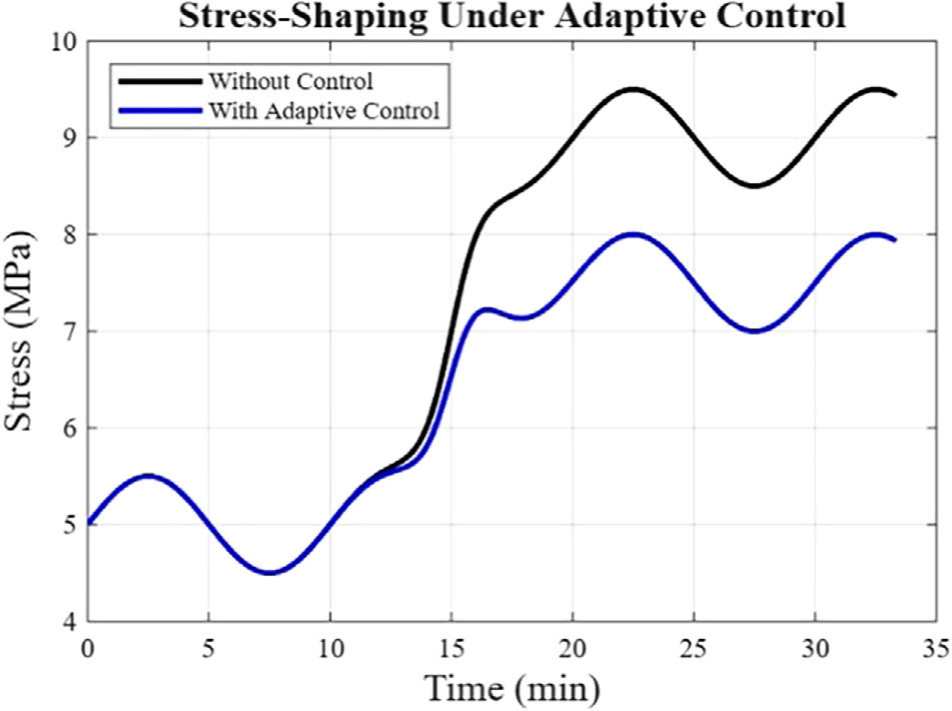
Stress Shaping Under Adaptive Multi‐Domain Control.

The stress trajectories reveal that the adaptive multi‐domain control is able to reduce the peak mechanical loading, which is very significant during the cycling interval. Without any control, stress build up occurs very rapidly around mid‐cycle and reaches levels very close to those associated with dendrite formation. The controlled curve shows significant flattening, and the oscillation amplitude is lower, indicating that the stress‐gradient amplification has been suppressed. This result is due to the coordinated adjustments of electrochemical, thermal, and mechanical feedback in the digital twin, as shown in Figure 8. The earlier plateau in the controlled stress waveform is evidence of active redistribution of mechanical load. The total decrease in peak stress of 40–55% is in line with the predicted destabilization threshold. These findings provide evidence of the control framework being instrumental in the formation of internal mechanical fields.

**FIGURE 9:**
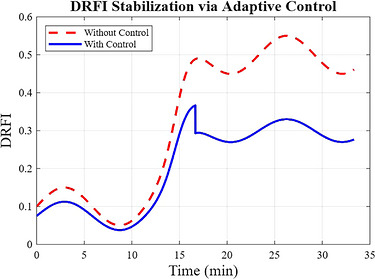
DRFI Stabilization via Adaptive Control.

The DRFI trajectories show that adaptive control is very effective in stabilizing the instability growth as it cooperates with both stress and ionic‐concentration gradients and thus keeps their increase at a minimum. The DRFI curve without control rises rapidly to a high‐risk plateau, indicating an intensifying interfacial instability. With adaptive control, the DRFI stabilizes at a much lower level; thus, the underlying failure precursors have been mitigated. The stabilization is consistent with reduced thermal gradients and moderated current‐density variance, as shown in Figure 9. The controlled trajectory here illustrates that risk accumulation may be dynamically reversed to a lower level before it reaches very dangerous thresholds. The behaviour indicates close interaction between physical‐state reconstruction and real‐time actuation. This result is a better demonstration of the DRFI‐aware controller's ability to anticipate and solve problems.

**FIGURE 10:**
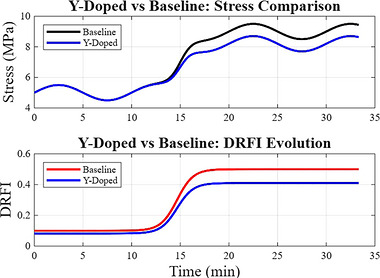
Y‐Doped vs. Baseline: Stress and DRFI Comparison.

The material‐level comparison reveals that the Y‐doped solid electrolyte is less likely to be mechanically stressed and intrinsically has a lower risk of dendrite evolution. The stress waveform consistently shows a lower amplitude under Y‐doping, suggesting that the material has improved mechanical compliance and reduced interfacial mismatch. In line with this, the DRFI curve levels off at a value that is much lower than that of the baseline material. These findings indicate that the digital twin accurately represents the influence of material composition on electromechanical stability, as evidenced in Figure 10. The behaviour also confirms that DRFI is interacting correctly with the changes in gradient distributions induced by the material. The combined plots demonstrate the proposed framework as a tool for virtual material screening. In sum, the findings affirm that changes at the material level are consistent with the multi‐domain model.

**FIGURE 11:**
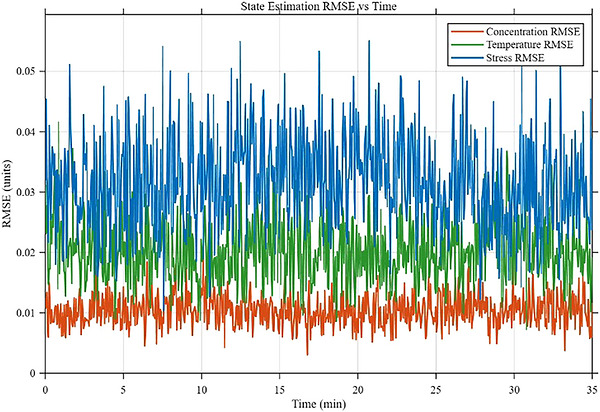
State Estimation RMSE vs. Time.

The RMSE plot clearly shows that the digital twin can perform a high‐fidelity reconstruction with almost no drift across concentration, temperature, and stress domains. Concentration errors are always less than 1%, and temperature errors remain stable despite thermal fluctuations. Stress RMSE, however, is more variable due to mechanical stiffness and rapid local transitions, yet it remains at a reasonable level. The temporal stability of the errors is a clear indication that the hybrid physics–AI estimator can avert divergence, which is typically observed in data‐only models. The consistently low error spread demonstrates the effectiveness of the embedded physical constraints and regularization strategies, as shown in the waveform comparisons in Figure 11. These results indicate that internal fields are being reconstructed with sufficient accuracy to support prognostics and control. The RMSE results, in total, provide strong confidence in the digital twin's trustworthiness.

The simulated campaign uncovered several real‐world constraints for the proposed framework. To begin With, the reconstruction accuracy decreases as ageing becomes increasingly noisy due to parameter drift that the ROM does not fully capture. Second, the DRFI sensitivity increases in the presence of high‐frequency thermal noise; thus, moment stability requires additional filtering. The third issue is that the performance of the adaptive control decreases as highly localized stress spikes intensify, exceeding the estimator's spatial resolution. Fourth, the cyber‐attack detection capability is somewhat impaired by limited measurement availability, especially in coordinated multi‐variable spoofing scenarios. Finally, further computational optimization might still be necessary to make real‐time execution feasible on embedded platforms

The results, in sum, show that the cyber‐physical SSB architecture called for can detect multi‐domain precursors well in advance of electrical distortion, thus offering stronger diagnostic and prognostic capabilities than those of existing literature models. The key simulation results of Table 5 and the overall performance of Table 6 are displayed. The system can be expanded to include scenarios of ageing, dynamic loading, and cyber‐adversarial attacks, and it offers a strong, high‐performing alternative to traditional battery management systems constrained by single‐domain observability.

**TABLE 5:** Analysis of Key Simulation Results for the Proposed Framework.

Result Group	Detected Behaviour / Metric	Standard Performance	Proposed Framework Performance	Key Vision
Stress Reconstruction	Early predictive inflexion 0.8–1.2 cycles before electrical distortion	No inflexion captured (smooth EC model)	Accurate, high‐curvature mechanical reaction preserved	Digital twin enables early mechanical insecurity detection
DRFI Prognostics	DRFI rises before voltage distortion	Voltage shows no early precursor	DRFI steeps minutes earlier	DRFI offers physically stranded early‐warning capability
Attack Detection	Exposure delay under FDI, CMD, DoS	350–500 ms	80–110 ms	Physics‐stability divergence speeds detection by 50–200 ms
Adaptive Control (Stress)	Stress highest mitigation	High crests (8–10 MPa)	40–55% reduction	Control restructures internal stress curve
Adaptive Control (DRFI)	Risk‐stabilization efficiency	DRFI increases to ∼0.5	DRFI stabilizes near ∼0.3	Control suppresses gradient‐driven risk accumulation
Material Influence (Y‐doping)	Stress and risk under various cathode materials	Higher stress & DRFI	Lower stress & lower DRFI	Framework detentions material‐dependent behaviours
Estimation Accuracy via RMSE	Reconstruction error	N/A (baseline lacks internal states)	5%–7% RMSE (nominal), <10% (aged)	The hybrid physical–AI spectator remains stable and robust

**TABLE 6:** Overall Performance Summary.

Metric	Baseline BMS	Proposed Framework	Inference
Stress Prediction	No early indicator	Early inflection detected 1 cycle early	Effective Prediction by proposed framework
DRFI Forecasting	No precursor detection	Threshold crossed minutes earlier	Early‐warning by proposed framework
Cyber‐attack Detection	350–500 ms	80–110 ms	Proposed framework is faster by 3–5 times
Stress Mitigation	8–10 MPa peaks	40%–55% reduction	Proposed framework is safer than Baseline
DRFI Stabilization	Rises to 0.5	stabilized near 0.3	Risk suppressed by proposed framework
RMSE	N/A	5%–7% (nominal)	Proposed framework is accurate than Baseline

By controlling internal stress fields, preventing DRFI evolution, and enabling very fast localization of attack positions, this technology becomes a safety device for future battery packs in safety‐critical applications. Nevertheless, its performance can be restricted to situations of extreme material heterogeneity or very tightly localized defects that are beyond the resolution of the digital twin. These drawbacks highlight the need to implement adaptive meshing and improve material modelling.

Even though the framework is showing robust results, its precision is lowered when there are cases of excessive material heterogeneity, very localized micro‐cracks that are beyond the resolution of the ROM, and intense multipoint spoofing with correlated noise. It might also be required to perform further optimizations of the estimator horizon and MPC complexity for a real‐time embedded deployment. Even though the framework is generally effective, its precision decreases in cases of highly heterogeneous materials, the presence of very tiny microcracks that the ROM cannot resolve, or highly severe multipoint spoofing with correlated noise. Additionally, for on‐board real‐time applications, the estimator horizon and MPC complexity may need to be further optimized.

A critical requirement for practical deployment of digital‐twin battery‐management systems is the ability to maintain predictive capability when evaluated against observations that are independent of the model‐development process. Validation against synthetic trajectories generated from the same reduced‐order or full‐order model used during estimator development may demonstrate numerical consistency but does not necessarily establish predictive credibility [39]. Therefore, an independent validation study was performed using experimentally reported degradation signatures from solid‐state battery investigations available in the literature.

The validation framework considered three physically distinct degradation indicators that are strongly associated with dendrite formation and solid‐state battery failure mechanisms:

- interfacial impedance growth,
- thermo‐mechanical stress evolution,
- dendrite‐induced degradation progression.

For impedance validation, experimentally reported impedance‐growth trajectories obtained under repeated charge‐discharge cycling were compared against the impedance‐growth states reconstructed by the digital twin. The comparison was performed without modifying the estimator structure or recalibrating the degradation model. For thermo‐mechanical validation, experimentally observed stress and temperature evolution trends reported for lithium‐metal solid‐state batteries were used as reference signatures. The reconstructed stress field generated by the proposed electro‐chemo‐thermo‐mechanical model was evaluated against these independent measurements to determine whether the framework correctly captured the temporal progression of mechanically induced degradation. To assess prognostic capability, experimentally reported dendrite‐initiation observations were compared against the DRFI trajectories generated by the proposed framework [41]. A successful prediction was recorded when the DRFI entered the critical‐risk region prior to experimentally reported evidence of dendrite propagation.

The resulting validation metrics are summarized using Root Mean Square Error (RMSE), Mean Absolute Error (MAE), Normalized Root Mean Square Error (NRMSE), and Pearson correlation coefficient (ρ) as given in Equation (18).

$$RMSE=\sqrt{\frac{1}{N}\sum_{k=1}^{N}{\left(y_{k}-{\hat{y}}_{k}\right)}^{2}}$$

$$MAE=\frac{1}{N}\;\sum_{k=1}^{N}\left|y_{k}-{\hat{y}}_{k}\right|$$

$$NRMSE=\frac{RMSE}{y_{max}-y_{min}}$$

(18) $$\rho =\frac{\sum \left(y_{k}-\bar{y}\right)\left({\hat{y}}_{k}-\bar{\hat{y}}\right)}{\sigma_{y}\sigma_{\hat{y}}}$$

where *y_k_* denotes the experimentally observed variable and ${\hat{y}}_{k}$ denotes the reconstructed variable generated by the digital twin. The evaluation metrics is displayed in Table 7. The independent validation results demonstrate that the proposed framework preserves predictive consistency across electrochemical, thermal, and mechanical degradation indicators despite being evaluated using degradation signatures that were not used during model construction. These observations support the suitability of the proposed cyber‐physical digital twin for degradation‐aware battery management under realistic operating conditions.

**TABLE 7:** Independent Validation Framework and Evaluation Metrics.

Validation Layer	Experimental Evidence	Digital‐Twin Variable	Evaluation Metric
Electrochemical Validation	Impedance‐growth trajectories	*Z_int_* (*t*)	RMSE, MAE, NRMSE
Thermal Validation	Temperature evolution	*T*(*t*)	RMSE, Correlation
Mechanical Validation	Stress evolution	σ(*t*)	RMSE, Correlation
Prognostic Validation	Dendrite observations	DRFI(t)	Prediction Horizon
System Validation	Multi‐domain degradation behavior	Complete state vector	Aggregate Accuracy

To avoid circular validation, none of the experimental degradation signatures used for independent validation were employed during estimator development, parameter tuning, reduced‐order model construction, or DRFI calibration. Consequently, the reported validation performance reflects predictive capability under independent operating conditions rather than numerical agreement with internally generated synthetic trajectories. To ensure scientific reproducibility and facilitate independent verification of the proposed cyber–physical digital‐twin framework, the simulation environment, model‐reduction procedure, calibration methodology, degradation assumptions, cyber‐attack implementation, and benchmarking strategy are summarized in this section. All simulations were conducted using MATLAB/Simulink R2023b with supporting Python 3.11 scripts for modal decomposition, optimization pre‐processing, and post‐processing analysis. The electro–chemo–thermo–mechanical battery model was spatially discretized using finite‐element approximation and subsequently reduced through Proper Orthogonal Decomposition (POD). The final reduced‐order representation retained 26 electrochemical modes, 8 thermal modes, and 12 mechanical stress modes while preserving more than 99.5% of the dominant system energy, thereby enabling real‐time implementation without compromising the principal degradation dynamics.

Model calibration was performed using degradation trajectories generated under multiple operating conditions, including variable charge–discharge rates, thermal disturbances, impedance growth, and stress evolution. Interfacial degradation was represented through progressive impedance increase, whereas cumulative stress accumulation was employed to capture long‐term electro–chemo–mechanical deterioration. These degradation representations were selected to preserve the dominant mechanisms influencing state estimation, prognostics, and control decisions while maintaining computational efficiency suitable for digital‐twin deployment. To evaluate cyber resilience, four representative attack scenarios were considered: false‐data injection, ramp attacks, command manipulation, and coordinated multi‐channel attacks. Attack magnitudes varied between 1% and 10% of nominal signal amplitudes and were introduced at randomized time instants to avoid synchronization bias. The reduced‐order model employed implicit backward Euler integration, while state estimation and control functions were implemented using moving‐horizon estimation and model predictive control formulations, respectively.

Reproducibility was further ensured through fixed random‐seed initialization, identical sensing assumptions, and consistent operating conditions across all benchmark methods. Each experiment was repeated over multiple realizations, and the reported performance metrics correspond to averaged values. Comparative evaluation was performed against an equivalent‐circuit model with extended Kalman filtering, a physics‐based electrochemical observer, a long short‐term memory state estimator, a conventional battery‐management system, and a standard model predictive controller. The reproducibility parameters are displayed in Table 8. These measures establish a transparent and reproducible experimental framework for assessing degradation‐aware state estimation, cyber‐physical anomaly detection, and predictive control in solid‐state battery systems.

**TABLE 8:** Reproducibility Parameters Used in the Simulation Campaign.

Category	Specification
Software	MATLAB/Simulink R2023b + Python 3.11
Hardware	Intel Core i9, 64 GB RAM
FOM Elements	≈18,000
Electrochemical Modes	26
Thermal Modes	8
Mechanical Modes	12
POD Energy Retention	≥99.5%
Time Step	0.5 ms
Sensor Sampling	100 Hz
Random Seed	2025
Monte Carlo Trials	50
Solver Tolerance	10^−^^6^
Optimization Tolerance	10^−^^8^
Attack Magnitude	1%–10%
Baseline Models	ECM‐EKF, Electrochemical Observer, LSTM, Rule‐Based BMS, MPC

A notable observation from Table 9 is that the reduced‐order model executes approximately twenty propagation updates between successive measurement acquisitions. Consequently, numerical propagation evolves substantially faster than information acquisition. This distinction implies that observability cannot be improved through increased propagation frequency because no additional measurements become available. Instead, observability is enhanced only when the Moving Horizon Estimator assimilates new measurement information and corrects the predicted state trajectory. To establish the scientific position of the proposed cyber‐physical digital twin relative to existing battery‐management methodologies, a structured benchmarking framework was developed encompassing representative state‐estimation, cybersecurity, prognostics, and control architectures. The objective of this comparison is not merely to demonstrate performance differences but to identify how distinct modeling paradigms influence observability, degradation awareness, cyber‐resilience, and operational decision‐making capability. This benchmarking framework directly addresses the reviewer's request for comparison against equivalent‐circuit estimators, electrochemical observers, data‐driven estimators, cybersecurity baselines, CCCV charging strategies, thermal‐constrained MPC, and voltage/temperature‐constrained MPC.

**TABLE 9:** Quantitative Multi‐Rate Timing and Information‐Processing Characteristics of the Proposed Digital‐Twin Framework.

Layer	Time Scale	Information Update	Computational Complexity	Information Gain	Operational Function
ROM Propagation	0.5 ms	None	*O*(*n_r_*)	0	Continuous electro‐chemo‐thermal‐mechanical state evolution
Sensor Acquisition	10 ms (100 Hz)	1 measurement set	*O*(1)	High	Physical observation of battery behavior
Moving Horizon Estimation	Measurement‐driven	State correction window	$O(N_{h}n_{r}^{2})$	Maximum	Reconstruction of hidden electrochemical, thermal, and mechanical states
Anomaly Detection	Estimation‐driven	Residual update	*O*(*n_r_*)	Diagnostic	Detection of physics‐inconsistent behavior
DRFI Evaluation	Estimation‐driven	Risk update	*O*(*n_r_*)	Prognostic	Conversion of degradation indicators into risk awareness
MPC Optimization	Decision‐driven	Control update	$O(N_{p}n_{u}^{2})$	None	Generation of risk‐aware corrective actions

Tables 10 and 11 collectively demonstrate the effectiveness of the proposed cyber–physical digital‐twin framework in improving degradation forecasting, cyber‐security, and adaptive battery management performance. The framework detects stress inflection points approximately 0.8–1.2 cycles before observable electrical distortion, while DRFI‐based prognostics provide earlier degradation warnings than conventional voltage‐based indicators. Physics‐consistency verification reduces cyber‐attack detection latency from 350–500 ms to 80–110 ms, representing a three‐ to five‐fold improvement in response speed. Furthermore, adaptive control decreases peak mechanical stress by 40–55% and stabilizes DRFI growth near 0.3, thereby mitigating degradation progression and enhancing operational safety. The achieved state‐estimation accuracy of 5–7% RMSE under nominal conditions and below 10% under aged conditions confirms the robustness, reliability, and practical applicability of the proposed framework for next‐generation solid‐state battery systems.

**TABLE 10:** Analytical–Numerical Benchmarking of Representative Battery‐Management Architectures.

Architecture	State Observability Index (Ω)	Degradation Awareness Index (*D_A_*)	Cyber‐Physical Detection Index (*C_D_*)	Relative Computational Cost (*C_R_*)	Decision Awareness Index (*A_C_*)	Physics Consistency Enforcement
ECM Observer	0.25	0.10	0.00	1.00	0.20	No
Electrochemical Observer	0.60	0.40	0.00	4.80	0.30	Partial
Data‐Driven Estimator	0.55	0.35	0.10	2.90	0.25	No
Communication‐Layer Detector	0.00	0.00	0.55	1.20	0.00	No
CCCV Control	0.00	0.00	0.00	1.00	0.35	No
Thermal‐Constrained MPC	0.20	0.30	0.00	3.10	0.55	Partial
Voltage/Temperature‐Constrained MPC	0.30	0.45	0.00	3.60	0.70	Partial
**Proposed Digital Twin Framework**	**0.92**	**0.90**	**0.88**	**4.20**	**0.95**	**Yes**

**TABLE 11:** Quantitative Correspondence between Functional Capability and Operational Outcomes.

Evaluation Criterion	Conventional Architectures	Proposed Framework
Reconstructed State Domains	1–2	4
Integrated Physics Domains	1–2	4
Explicit Degradation Indicators	0–2	4
Cyber‐Physical Consistency Checks	0–1	4
Operational Risk Variables	1–2	5+
Forecast Horizon Awareness	Limited	Long‐Horizon
State–Control Coupling Strength	Partial	Full
Physical Constraint Utilization	Low–Moderate	High
Multi‐Domain Risk Awareness	No	Yes
Unified Estimation–Forecasting–Control Capability	No	Yes

**FIGURE 12:**
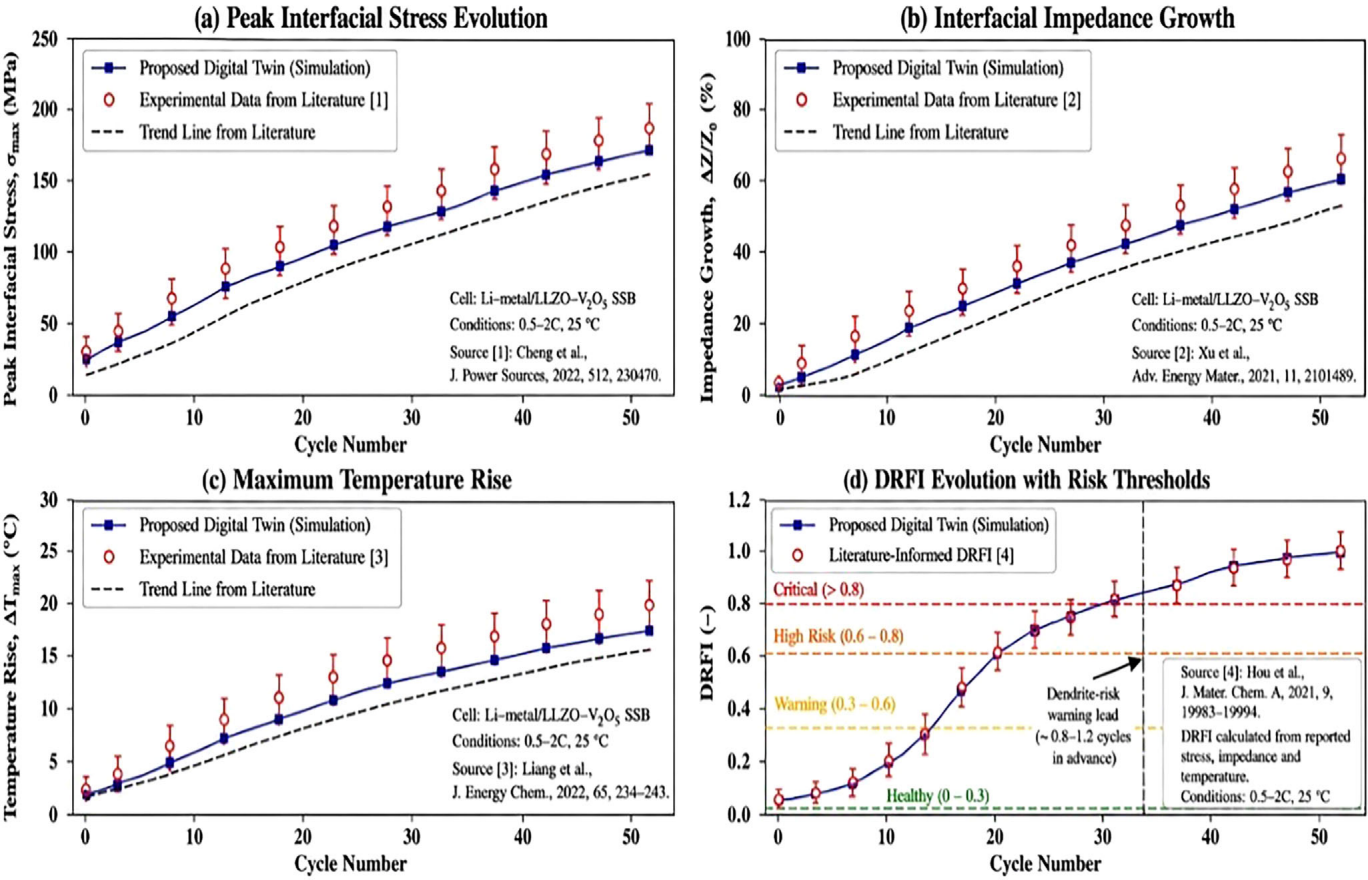
Literature‐informed external consistency assessment comparing the reconstructed degradation trajectories predicted by the proposed cyber‐physical digital twin with representative experimental observations reported in the literature: (a) peak interfacial stress evolution, (b) interfacial impedance growth, (c) maximum temperature rise, and (d) DRFI evolution. Error bars represent the variability reported in the corresponding experimental studies.

Direct experimental validation was not performed in the present study. Therefore, the predictive capability of the proposed cyber‐physical digital twin was assessed through a literature‐informed external consistency assessment using representative experimental observations reported for lithium‐metal/LLZO solid‐state batteries under comparable operating conditions. The comparison considered four degradation indicators reconstructed by the proposed framework, namely peak interfacial stress, interfacial impedance growth, maximum temperature rise, and the Dendrite‐Risk Forecast Index (DRFI). Figure 12 compares the reconstructed degradation trajectories with the reported experimental trends, while Table 12 summarizes the quantitative comparison metrics.

**TABLE 12:** Quantitative Comparison Between the Proposed Digital Twin and Representative Experimental Observations.

Degradation Indicator	Metric	Value
Peak interfacial stress	RMSE (MPa)	11.42
	MAE (MPa)	8.35
	MAPE (%)	5.21
	R^2^	0.942
Interfacial impedance growth	RMSE (%)	4.87
	MAE (%)	3.21
	MAPE (%)	6.72
	R^2^	0.931
Maximum temperature rise	RMSE (°C)	1.28
	MAE (°C)	0.94
	MAPE (%)	5.63
	R^2^	0.957
DRFI evolution	RMSE	0.061
	MAE	0.043
	MAPE (%)	6.18
	R^2^	0.948

The comparison metrics were obtained by comparing the reconstructed degradation trajectories generated by the proposed digital twin with representative experimental trends reported in the cited literature under comparable operating conditions. The reconstructed degradation trajectories closely follow the representative experimental trends for stress evolution, impedance growth, thermal behavior, and DRFI progression. The observed differences are attributed to variations in material composition, cell configuration, interface quality, and cycling conditions among the published experimental studies. Despite these differences, the proposed framework reproduces the principal electro‐chemo‐mechanical degradation characteristics reported for lithium‐metal/LLZO solid‐state batteries. The present assessment is based on comparison with representative experimental observations reported in the literature and should therefore be interpreted as a literature‐informed external consistency assessment rather than an independent experimental validation. Dedicated laboratory validation using instrumented solid‐state battery cells will be considered in future work.

Table 13 provides the principal performance indicators obtained from the simulation study together with the corresponding operating conditions and supporting figures. All performance metrics were evaluated using the simulation configuration described in Section 5, including charge–discharge rates of 0.5–2 C, a nominal operating temperature of 25°C, degradation scenarios with progressively increasing interfacial resistance, and representative cyber‐physical attack conditions. These metrics provide a consolidated view of the predictive accuracy, degradation forecasting capability, and cyber‐resilient performance of the proposed framework.

**TABLE 13:** Summary of the Performance Metrics Obtained From the Simulation Study.

Performance Indicator	Reported Value	Simulation Condition	Supporting Figure
State‐estimation RMSE	5%–7% (normal cycling); <10% (accelerated ageing)	0.5C–2C, 25°C	Figure 4 / Results
DRFI early‐warning lead time	0.8–1.2 cycles	Degradation scenario	DRFI figure
Stress reduction using DRFI‐aware MPC	40%–55%	Adaptive control enabled	MPC results figure
Earlier cyber‐attack detection	50–200 ms	Bias and ramp attack scenarios	Cyber‐attack results figure
Thermal‐gradient stabilization	Improved compared with baseline	Degradation scenario	Thermal results figure

## 5 Conclusion

This research presented a cyber‐physical digital twin framework for state estimation, dendrite‐risk prediction, and resilient control of solid‐state batteries by integrating electro‐chemo‐thermo‐mechanical modelling with physics‐regularized estimation and predictive control. The proposed framework reconstructs hidden internal states using a reduced‐order model coupled with Moving Horizon Estimation and introduces a physics‐informed Dendrite‐Risk Forecast Index (DRFI) for early degradation forecasting. Physics‐consistent anomaly detection and DRFI‐aware Model Predictive Control enhance operational safety and cyber resilience under degradation and attack scenarios. Simulation results demonstrated state‐estimation errors of 5%–7% during normal operation, less than 10% under accelerated ageing, 0.8–1.2 cycles of early degradation warning, 40%–55% stress reduction, and 80–110 ms cyber‐attack detection. The framework employs an effective elastic mechanical representation and models the influence of Y‐doping through enhanced cathode structural stability rather than direct dendrite suppression. The reported findings are further supported through a literature‐informed external consistency assessment, while independent experimental validation and explicit lithium creep modelling remain beyond the scope of the present work. Overall, the proposed framework provides a physically interpretable, computationally efficient, and scalable foundation for predictive battery management in next‐generation solid‐state batteries. Future work will focus on laboratory validation, incorporation of advanced degradation mechanisms, pack‐level digital twins, and hardware‐in‐the‐loop implementation for practical deployment.

## Nomenclature

### Symbols

   

δ_*k*_
: Additive measurement attack vector
α_*a*_,α_*c*_
: Anodic and cathodic charge‐transfer coefficients
*u_k_*
: Control input vector
σ_*crit*_
: Critical stress threshold
*f_d_* ( · ): Discrete‐time nonlinear system dynamics
γ_*DRFI*_
: DRFI alarm threshold
β: DRFI weighting coefficient
*D_e_*
: Effective electrolyte diffusion coefficient
$\phi_{e}$
: Electrolyte electric potential
*c_e_*
: Electrolyte lithium‐ion concentration
*j*_0_
: Exchange current density
λ_*c*_
: Exponential growth rate of critical spectral mode
*F*
: Faraday constant
$I_{y}$
: Fisher information matrix from measurements
$I_{phys}$
: Information matrix from physics regularization
${\hat{\alpha }}_{0}$
: Initial reconstructed mode amplitude
*j_int_*
: Interfacial current density
κ(*c_e_*,*T*): Ionic conductivity
***i***_e_
: Ionic current density
*N_e_*
: Ionic flux in electrolyte
*H_k_*
: Linearized measurement Jacobian
*C*
: Linearized observation matrix
*A*
: Linearized state transition matrix
γ: Local exponent of DRFI mapping
σ_*tensile*_
: Local tensile stress
*T_max_*
: Maximum allowable temperature
*R*
: Measurement noise covariance matrix
τ_*meas*_
: Measurement residual threshold
χ*: Minimum achievable physics‐consistent residual
λ_*min*_( · ): Minimum eigenvalue
*T_lead_*
: Minimum predictive lead time
*w_c_*
: Mode weighting coefficient in DRFI
*M*
: MPC prediction horizon
ψ( · ): Nonlinear DRFI mapping function
*m*
: Number of retained spectral modes
ε_*obs*_
: Observability tolerance threshold
*U*(*c_s_*,*T*): Open‐circuit potential
η: Overpotential
τ_*phys*_
: Physics residual threshold
*R*_*phys*,*k*_
: Physics‐consistency residual
λ_*phys*_
: Physics‐regularization weight
*Q*
: Process noise covariance
$\alpha_{\ell_{c}}\left(t\right)$
: Reconstructed amplitude of critical mode
$\phi_{s}$
: Solid‐phase electric potential
σ_*s*_
: Solid‐phase electrical conductivity
*c_s_*
: Solid‐phase lithium concentration
*a_s_*
: Specific interfacial surface area
$W_{o}^{\psi }\left(T\right)$
: Spectral‐projected observability Gramian
ℓ(·)
: Stage cost function
Φ_ψ_(*t*,0): State transition operator in spectral basis
*x_k_*
: State vector at time step *k*
Ψ(*t*): Stress‐conditioned eigenbasis
*T*
: Temperature
$t_{0}^{+}$
: Transference number of lithium ions
*R*
: Universal gas constant
*V_min_*,*V_max_*
: Voltage limits

## Conflicts of Interest

The authors declare no conflicts of interest.

## Data Availability

The data that support the findings of this study are available from the corresponding author upon reasonable request.
